# *MYB44* competitively inhibits the formation of the *MYB340*-*bHLH2*-*NAC56* complex to regulate anthocyanin biosynthesis in purple-fleshed sweet potato

**DOI:** 10.1186/s12870-020-02451-y

**Published:** 2020-06-05

**Authors:** Zeng-Zheng Wei, Kang-Di Hu, Dong-Lan Zhao, Jun Tang, Zhong-Qin Huang, Peng Jin, Yan-Hong Li, Zhuo Han, Lan-Ying Hu, Gai-Fang Yao, Hua Zhang

**Affiliations:** 1 School of Food and Biological Engineering, Hefei University of Technology, Hefei, 230009 China; 2 Xuzhou Institute of Agricultural Sciences of the Xuhuai District of Jiangsu Province, Xuzhou, 221131 China; 3 Department of Ecology and Environment of Anhui Province, Hefei, 230061 China

**Keywords:** Sweet potato (*Ipomoea batatas*), Anthocyanin biosynthesis, *IbMYB340*, *IbMYB44*, *IbNAC56*, Regulatory complex, Repressor

## Abstract

**Background:**

Anthocyanins, which have important biological functions and have a beneficial effect on human health, notably account for pigmentation in purple-fleshed sweet potato tuberous roots. Individual regulatory factors of anthocyanin biosynthesis have been identified; however, the regulatory network of anthocyanin biosynthesis in purple-fleshed sweet potato is unclear.

**Results:**

We functionally determined that *IbMYB340* cotransformed with *IbbHLH2* in tobacco and strawberry receptacles induced anthocyanin accumulation, and the addition of *IbNAC56a* or *IbNAC56b* caused increased pigmentation. Furthermore, we confirmed the interaction of IbMYB340 with IbbHLH2 and IbNAC56a or IbNAC56b via yeast two-hybrid and firefly luciferase complementation assays; these proteins could form a MYB340-bHLH2-NAC56a or MYB340-bHLH2-NAC56b transcriptional complex to regulate anthocyanin biosynthesis by binding to the *IbANS* promoter rather than the *IbUFGT* promoter. Furthermore, it was found by a transient expression system in tobacco leaves that *IbMYB44* could decrease anthocyanin accumulation. Moreover, the interaction of IbMYB44 with IbMYB340 and IbNAC56a or IbNAC56b was verified. This result suggested that *IbMYB44* acts as a repressor of anthocyanin in sweet potato.

**Conclusions:**

The repressor *IbMYB44* affected anthocyanin biosynthesis by competitively inhibiting the *IbMYB340*-*IbbHLH2*-*IbNAC56a* or *IbMYB340*-*IbbHLH2*-*IbNAC56b* regulatory complex formation. Overall, the present study proposed a novel regulatory network whereby several vital TFs play key roles in regulating anthocyanin biosynthesis, and it provides strong insight into the potential mechanism underlying anthocyanin biosynthesis in sweet potato tuberous roots with purple color.

## Background

Sweet potato (*Ipomoea batatas*) is well known for its abundant nutritional value and is the fourth most important crop species in China [1, 2]. The colors of its storage roots mainly include white, yellow, orange and purple. Among the different colored sweet potato types, purple-fleshed sweet potato is widely popular for its high anthocyanin contents in its storage roots. Anthocyanin is now recognized as the most important secondary metabolite in plants and has important biological functions, including disease resistance, UV radiation protection and defense against herbivores and pathogens [3, 4]. Moreover, the addition of anthocyanins in human recipes has a beneficial effect on the prevention of cancer and diabetes and on cardiovascular and neuronal illnesses [5].

The anthocyanin biosynthesis pathway has been extensively documented in different plant species and includes several key structural genes, such as phenylalanine ammonia lyase (PAL), flavanone 3-hydroxylase (F3H), dihydroflavonol 4-reductase (DFR), anthocyanidin synthase (ANS), UDP-glucose flavonoid 3-O-glucosyltransferase (UFGT) and glutathione S-transferase (GST) [6–8]. Transcription factors, especially the MBW complex, have been verified to be involved in synergistically regulating anthocyanin synthesis [9]. Among them, R2R3-MYBs particularly make a major contribution to anthocyanin biosynthesis [10]. For example, *AtMYB75*, *AtMYB113* and *AtMYB114* in *Arabidopsis* [11]; *MdMYB10* and *MdMYB110a* in apple [12, 13]; *PyMYB10* and *PyMYB114* in pear [14, 15]; *LcMYB5* in litchi [16]; *PaMYB10* in apricot [17]; and *FaMYB10* in strawberry [18] have been reported to be involved in anthocyanin biosynthesis as activators. In addition to MYB activators, R3-MYB and some R2R3-MYB repressors, including the chrysanthemum *CmMYB#7* (an R3-MYB), strawberry *FaMYB1* and *FaMYB44.1*, peach *PpMYB18* and potato *StMYB44* (R2R3-MYBs) ones, have also been identified as participating in the flavonoid biosynthetic pathway [19–22].

In addition to MYB TFs, other TFs are also involved in anthocyanin biosynthesis. NAC (NAM, ATAF1/2, and CUC2) TFs have also been widely documented to participate in multiple biological processes, such as plant development, disease resistance, and the abiotic stress response [23, 24]. For secondary metabolism, many NAC TFs have been verified to be involved in the phenylpropanoid pathway, thereby regulating lignin biosynthesis [25, 26]; it was reported that *ANAC032* acts as a repressor of anthocyanin accumulation in *Arabidopsis thaliana* during stress conditions [24]. Moreover, *ANAC078* and *PpBL* are characterized as activators of anthocyanin biosynthesis under certain conditions [27, 28].

In purple-flesh sweet potato, individual regulatory factors of anthocyanin biosynthesis have been identified [29, 30]. *IbMYB1* can promote the expression of anthocyanin-related genes specifically in purple-fleshed sweet potato [30]. Another factor, *IbWD40*, was documented to have a positive correlation with anthocyanin contents in different sweet potato cultivars, suggesting that *IbWD40* plays a key role in anthocyanin biosynthesis in purple-fleshed sweet potato [29]. However, additional evidence could not be provided due to the slow process brought forth by the complexity of the sweet potato genome, which is hexaploid (2n = 6x = 90) and highly polymorphic [31]. Thus, whether and how other NAC TFs and repressor MYBs participate in anthocyanin biosynthesis are unclear, and the molecular regulatory network of anthocyanin biosynthesis in purple-fleshed tuberous roots has rarely been reported.

In this study, four candidate TFs, *IbMYB340*, *IbMYB44, IbNAC56a* and *IbNAC56b,* were screened by bioinformatics and RT-qPCR analysis in sweet potatoes of different colors, and the gene functions were identified by transient expression assays in tobacco and strawberry receptacles. Further analysis indicated that *IbMYB340* contributes greatly to anthocyanin accumulation and could also form a regulatory complex with cofactors *IbbHLH2*, *IbNAC56a* and *IbNAC56b* to regulate anthocyanin biosynthesis by binding to the *IbANS* promoter, rather than the *IbUFGT* promoter, whereas *IbMYB44* acts as a negative regulator and could interact with *IbMYB340* and *IbNAC56a* or with *IbNAC56b* to regulate anthocyanin biosynthesis in sweet potato, as reflected by yeast two-hybrid and firefly luciferase complementation assays. Our study reveals a possible underlying mechanism of anthocyanin biosynthesis in purple-fleshed sweet potato, which will provide us with a potential understanding of the regulatory network of sweet potato roots with different colors.

## Results

### Characterization of *IbMYB* and *IbNAC* anthocyanin-related genes in sweet potato

**Fig. 1:**
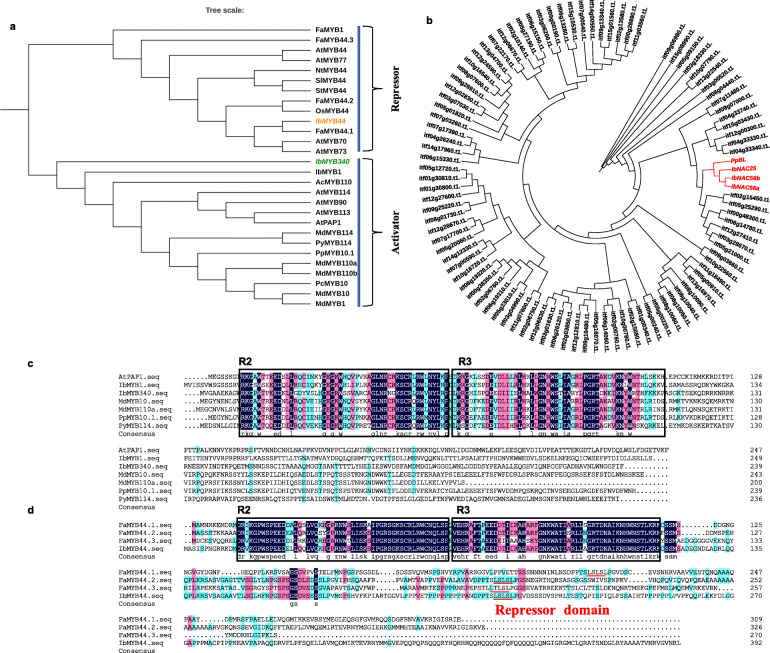
Phylogenetic analysis and multiple sequence alignments of NACs and MYBs. **a** Phylogenetic tree derived from the amino acid sequences of R2R3-MYBs in sweet potato and other species. *IbMYB340* and *IbMYB44* are marked by orange or green letters, respectively. The analysis of evolutionary history inferred using the neighbor-joining method was conducted by MEGA 7. **b** Phylogenetic analysis of 98 *IbNAC* genes in sweet potato and other species. In addition, *IbNAC25*, *IbNAC56a*, *IbNAC56b* and *PpBL* are highlighted with red letters. **c**-**d** The protein sequences of R2R3-MYBs in sweet potato and other species were used to perform the sequence alignment. In addition, the R2 and R3 domains are labeled, and the LxLxL negative repressor motif is highlighted with a red underscore

In previous reports, TFs *AtMYB75* (*AtPAP1*) in *Arabidopsis thaliana* and *MdMYB10* (DQ267897) and *PyMYB114* (ASY06612.1) in non-model species were found to be R2R3-type MYBs, which may work together with other TFs in regulating anthocyanin biosynthesis [11, 12, 15]. Here, we found that the Itf12g05820.t1 gene was highly homologous with *AtPAP1* (AT1G56650) by BLAST alignment against the sweet potato genome database. It was named *IbMYB340* after multiple sequence alignment revealed its similarity with other typical R2R3-MYB TFs in other species. In addition, *IbMYB44* was identified from *FaMYB44.1* by homologous sequence alignment; *FaMYB44* is a transcriptional repressor that negatively regulates sucrose accumulation in strawberry receptacles through interplay with *FaMYB10* [19]. Phylogenetic analysis indicated that *IbMYB340* belongs to the activator clade, together with other known MYB transcription factors with the R2R3 domain (Fig. 1a). By contrast, TF *IbMYB44*, which harbors the transcriptional repressor domain LxLxL (Fig. 1d), was phylogenetically related to *FaMYB44.1* (Fig. 1a), which acts as a transcriptional repressor in strawberry [19]. Thus, we speculated that these two MYB TFs may regulate anthocyanin biosynthesis in sweet potato.

We retrieved sequence information for 98 NAC TFs containing a NAC domain in the N-terminal region from the sweet potato genome database. Amino acid sequences of NACs in sweet potato and peach *PpBL* (ALK27819.1), which can promote the activity of *PpMYB10.1* by acting as a heterodimer with *PpNAC1*, resulting in anthocyanin accumulation in tobacco leaves [28], were used to construct a phylogenetic tree (Fig. 1b). Three NAC genes, *IbNAC56a*, *IbNAC56b,* and *IbNAC25*, were the candidates according to the phylogenetic analysis results.

**Fig. 2:**
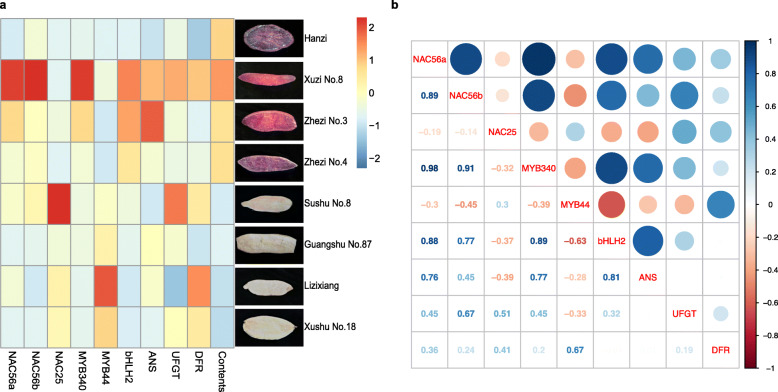
**a** The expression levels of *IbNAC56a*, *IbNAC56b*, *IbNAC25*, *IbMYB340*, *IbMYB44*, *IbbHLH2*, *IbANS*, *IbUFGT* and *IbDFR*, and anthocyanin contents in the tuberous roots of different sweet potato cultivars (‘Hanzi’, ‘Xuzi No. 8’, ‘Zhezi No. 3’, ‘Zhezi No. 4’, ‘Sushu No. 8’, ‘Guangshu No. 87’ and ‘Lizixiang’, ‘Xushu No. 18’). **b** Correlation analysis among the gene expression levels of *IbNAC56a*, *IbNAC56b*, *IbNAC25*, *IbMYB340*, *IbMYB44*, *IbbHLH2*, *IbANS*, *IbUFGT* and *IbDFR*. Pearson’s correlation coefficients for the data were analyzed using R scripts

Then, we quantified the transcript abundance of candidate genes in tuberous roots of different sweet potato cultivars, and the sectional drawings for tuberous roots of different cultivars sweet potatoes were shown in Fig. 2a. The *IbNAC56a* and *IbNAC56b* expression levels were significantly higher in purple-fleshed sweet potato cultivars ‘Xuzi No. 8’ and ‘Zhezi No. 3’ than in yellow-fleshed sweet potatoes ‘Sushu No. 8’ and ‘Guangshu No. 87’ or in white-fleshed sweet potatoes ‘Lizixiang’ and ‘Xushu No. 18’, whereas the expression pattern of *IbNAC25* was the opposite. Therefore, *IbNAC56a* and *IbNAC56b* were selected for subsequent experiments (Fig. 2a).

Moreover, the transcript abundance of *IbMYB340* and *IbbHLH2* was significantly higher in ‘Xuzi No. 8’ and ‘Zhezi No. 3’ than in the other cultivars, while the expression of *IbMYB44* was maintained at lower levels in purple-fleshed sweet potatoes than in yellow−/white-fleshed sweet potatoes. Furthermore, ‘Lizixiang’ and ‘Xushu No. 18’ presented higher transcript levels of *IbMYB44* than did ‘Sushu No. 8’ and ‘Guangshu No. 87’. The expression level of *IbANS* was also higher in the purple-fleshed cultivars except for ‘Hanzi’, while the yellow−/white-fleshed cultivars showed relatively low gene expression. However, the expression of *IbUFGT* and *IbDFR* showed no obvious difference (Fig. 2a).

Further, we analyzed the correlation among anthocyanin biosynthesis-related gene expression levels in the different sweet potato cultivars. As shown in Fig. 2c, the expression of *IbNAC56a, IbNAC56b, IbbHLH2* and *IbANS* displayed noticeably positive correlations with *IbMYB340* expression, and the correlation coefficients ranged from 0.77 to 0.98. However, the expression of *IbMYB44* had negative correlations with the expression of *IbMYB340, IbNAC56a, IbNAC56b, IbbHLH2, IbANS* and *IbUFGT*, suggesting that there might be an opposite effect of *IbMYB340* and *IbMYB44.* In addition, *IbNAC25* was negatively correlated with other factors (except *IbMYB44*, *IbDFR* and *IbUFGT*).

### Anthocyanin was induced by cotransforming*IbMYB340*, *IbbHLH2* and *IbNAC56a* or *IbNAC56b* in tobacco leaves

**Fig. 3:**
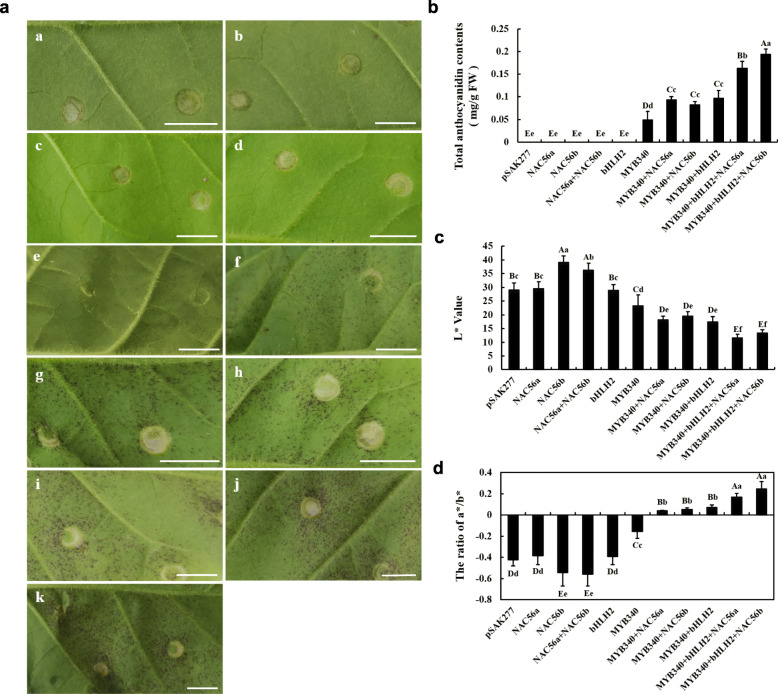
Functional analysis of the *IbMYB340, IbbHLH2* and *IbNAC56a* or *IbNAC56b* genes using a transient expression assay in tobacco leaves. **a** The phenotype of tobacco leaves on the 7th day after infiltration: a, pSAK277; b, *IbNAC56a*; c, *IbNAC56b*; d, *IbNAC56a* + *IbNAC56b*; e, *IbbHLH2*; f, *IbMYB340*; g, *IbMYB340* + *IbNAC56a*; h, *IbMYB340* + *IbNAC56b*; i, *IbMYB340* + *IbbHLH2*; j, *IbMYB340* + *IbbHLH2* + *IbNAC56a*; and k, *IbMYB340* + *IbbHLH2* + *IbNAC56b*. Scale bar = 1 cm. **b** Total anthocyanin contents in transformed leaves of tobacco. FW, fresh weight. **c**-**d** The color parameter L* and a*/b* ratio values were measured with a Minolta Chroma Meter and were used to indicate phenotypic changes. The values are presented as the means ± SDs (*n* = 3), and the uppercase and lowercase letters represent significant differences at *P* < 0.01 or *P* < 0.05, respectively

The results of a functional analysis of *IbMYB340*, *IbbHLH2* and *IbNAC56a* or *IbNAC56b* using a transient expression assay in tobacco leaves are shown in Fig. 3a. No anthocyanin accumulation was detected at the injection areas of tobacco leaves when transformed with *IbNAC56a*, *IbNAC56b* or *IbbHLH2* alone, while slight pigmentation appeared with *IbMYB340* injected alone. However, an enhanced color was visible at injection regions 7 day after transformation with both *IbMYB340* and *IbNAC56a/IbNAC56b* or *IbbHLH2*. When *IbNAC56a* or *IbNAC56b* was cotransformed with *IbMYB340* and *IbbHLH2*, obvious intense pigmentation was detected. The quantification of total anthocyanin contents indicated that anthocyanin biosynthesis was induced by cotransformation of three TFs, which was more than that from two TFs or *IbMYB340* alone, whereas tobacco leaves did not show any visible anthocyanin accumulation after transformation with the empty vector or with *IbNAC56a* or *IbNAC56b* alone (Fig. 3b). We next analyzed the color of the tobacco leaves. The L* values apparently declined when the pigmentation appeared; by contrast, the a*/b* ratio significantly increased (Fig. 3c-d).

### Heterologous overexpression of *IbMYB340, IbbHLH2* and *IbNAC56a* or *IbNAC56b* induces anthocyanin biosynthesis in strawberry receptacles

**Fig. 4:**
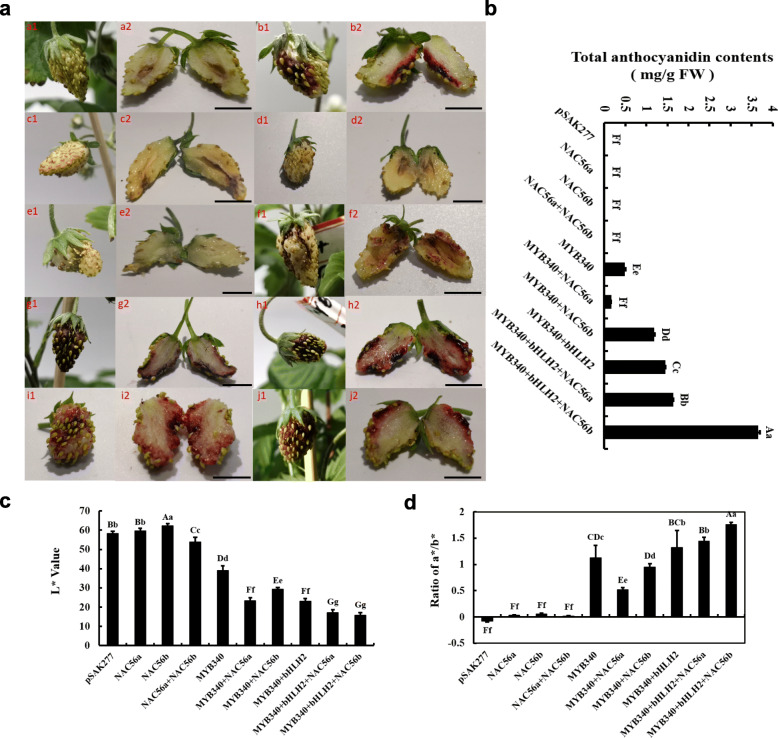
Transient expression of *IbMYB340* and *IbNACs* in strawberry receptacles. **a** The phenotype of strawberry receptacles: a, pSAK277; b, *IbMYB340*; c, *IbNAC56a*; d, *IbNAC56b*; e, *IbNAC56a* + *IbNAC56b*; f, *IbMYB340* + *IbNAC56a*; g, *IbMYB340* + *IbNAC56b*; h, *IbMYB340* + *IbbHLH2*; i, *IbMYB340* + *IbbHLH2* + *IbNAC56a*; and j, *IbMYB340* + *IbbHLH2* + *IbNAC56b*. Scale bar = 1.5 cm. **b** Anthocyanin contents in transformed strawberry fruits. FW, fresh weight. **c**-**d** The color parameter L* and a*/b* ratio values indicate phenotypic changes. The values are presented as the means ± SDs (n = 3), and the uppercase and lowercase letters represent significant differences at *P* < 0.01 or *P* < 0.05, respectively

To further confirm the roles of *IbMYB340* and *IbNAC56a* or *IbNAC56b* in anthocyanin biosynthesis, we cotransformed *IbMYB340* and *IbNAC56a* or *IbNAC56b* in strawberry receptacles via agroinfiltration, and diploid strawberry (*Fragaria vesca*) ‘Yellow Wonder’ 5AF7 was used for the transient transformation experiment. The transient expression assays in strawberry receptacles showed that coinfiltration of *IbMYB340* with any of the other candidate TFs resulted in apparent pigmentation accumulation 7 day after transformation, while the coinfiltration of three TFs caused deeper red pigmentation than did two TFs or *IbMYB340* alone. However, no pigmentation was observed when the empty vector was infiltrated; *IbNAC56a* or *IbNAC56b* were infiltrated separately; or *IbNAC56a* and *IbNAC56b* were coinfiltrated. In addition, pigmentation was also visible in the injection region of *IbMYB340* alone (Fig. 4a). Measurements of the induced anthocyanin are shown in Fig. 4b. The total anthocyanin contents of the three TFs cotransformed were significantly higher than those when the two TFs or one TF alone were cotransformed. The L* and a*/b* values of the injection regions of the strawberry receptacles declined or increased sharply when pigmentation appeared and stayed at significantly relatively low or high levels, respectively (Fig. 4c-d). Heterologous overexpression systems in strawberry receptacles presented phenotypic changes similar to those presented in the tobacco leaves in this study. We observed great anthocyanin pigmentation when *IbMYB340* was cotransformed with *IbbHLH2* and *IbNAC56a* or *IbNAC56b* together.

### Expression patterns of anthocyanin-related genes in strawberry receptacles

**Fig. 5:**
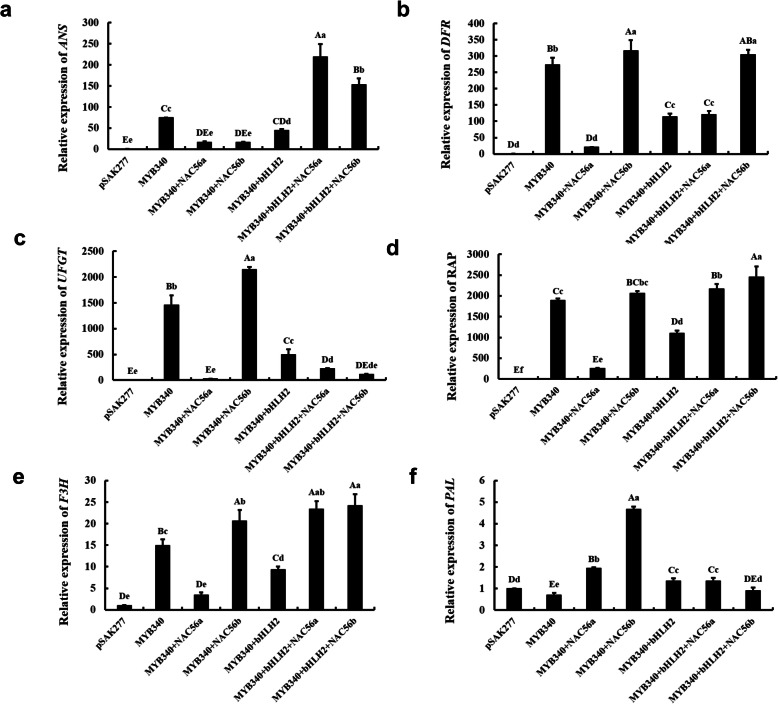
Expression levels of anthocyanin biosynthesis- and vacuolar transport-related genes in induced-color strawberry receptacles from Fig. 4. **a**-**f**: *FvANS*, *FvDFR*, *FvUFGT*, *FvRAP*, *FvF3H*, and *FvPAL.* The values are presented as the means ± SDs (n = 3), and the uppercase and lowercase letters represent significant differences at *P* < 0.01 or *P* < 0.05, respectively

To explore the regulatory models of the TFs *IbMYB340*, *IbbHLH2* and *IbNAC56a* or *IbNAC56b*, several critical anthocyanin-related genes, *FvPAL*, *FvF3H*, *FvANS*, *FvDFR*, *FvUFGT* and *FvRAP* were analyzed in induced-color strawberry receptaclesby RT-qPCR. *FvANS* expression was greatly increased when *IbMYB340*, *IbbHLH2* and *IbNAC56a* or *IbNAC56b* were cotransformed (*P* < 0.01, Fig. 5a). Interestingly, the transformation of *IbMYB340* alone showed a higher *FvANS* expression level than did cotransformation of *IbMYB340* and of *IbbHLH2* and *IbNAC56a* or *IbNAC56b* (*P* < 0.01 or *P* < 0.05, respectively). For *FvDFR*, transformation of TFs, including *IbNAC56b* or *IbMYB340* alone, resulted in noticeably gene expression levels that were higher than those from the other TF combinations (*P* < 0.01), while the additional *IbbHLH2* with *IbMYB340* and *IbNAC56a* cotransformation caused an obvious increase in *FvDFR* expression levels (*P* < 0.01, Fig. 5b). Figure 5d-e illustrates similar changes in the expression levels of *FvRAP* and *FvF3H*; these two genes were highly expressed in the cotransformation with different TFs or *IbMYB340* alone, except for *IbMYB340* + *IbNAC56a* (*P* < 0.01). In addition to *IbMYB340* + *IbNAC56a*, *IbMYB340* + *IbbHLH2* also maintained a relatively low expression level of *FvRAP* and *FvF3H* compared with that of other cotransformation types, excluding the empty vector pSAK277 (*P* < 0.01). From Fig. 5c, we can see that *FvUFGT* was significantly expressed in response to the cotransformation of *IbMYB340* + *IbNAC56b* or *IbMYB340* alone (*P* < 0.01), whereas other cotransformation types seemed not to activate the transcription of *FvUFGT*. In addition, it seemed that *FvPAL* could not be activated by different cotransformation types except for the cotransformation of *IbMYB340* and *IbNAC56b* (Fig. 5f). As mentioned above, almost all the genes involved in the anthocyanin biosynthetic pathway and vacuolar transport were highly expressed in the abovementioned cotransformed strawberries, especially when *IbMYB340*, *IbbHLH2,* and *IbNAC56a* or *IbNAC56b* were cotransformed. This result suggested that the *IbMYB340*, *IbbHLH2* and *IbNAC56a* or *IbNAC56b* genes might regulate or coregulate anthocyanin synthesis by forming a regulatory complex.

### The regulatory complex *MYB340-bHLH2-NAC56* promotes anthocyanin biosynthesis by binding to the *IbANS* promoter

**Fig. 6:**
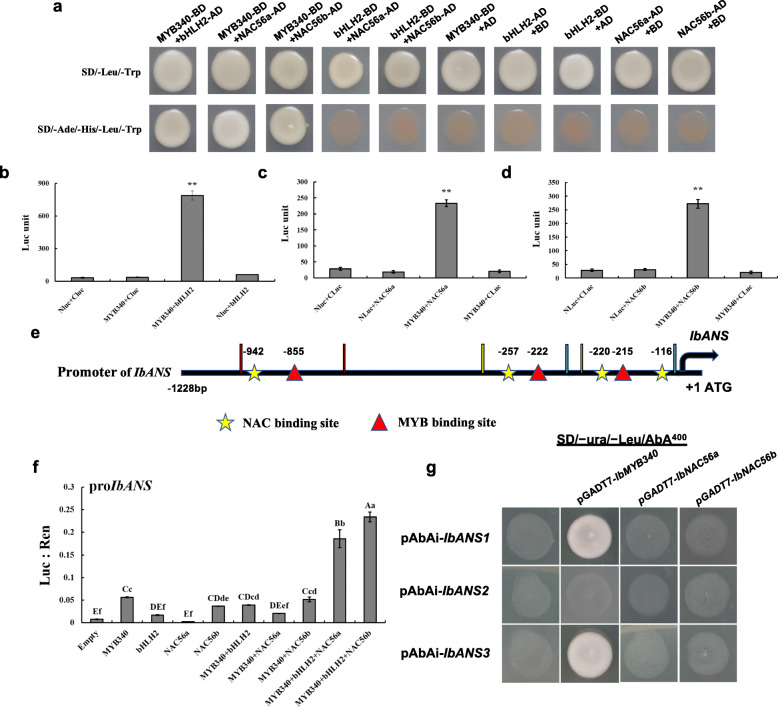
Interaction verification of IbMYB340 with IbbHLH2, IbNAC56a or IbNAC56b and coregulatory site evaluation of the *IbANS* promoter. **a** Verification of the interaction of IbMYB340 with IbbHLH2, IbNAC56aor IbNAC56b via Y2H assays. **b**-**d** Verification of the interaction between IbMYB340 and IbNAC56aor IbNAC56b via firefly luciferase complementation assays. ** indicates a significant difference at the *P* < 0.01 level. **e** Schematic of the *IbANS* promoter. The prediction of the cis-acting elements in the 1228-bp promoter region of *IbANS* was performed using the PlantCare database [32]. **f** Validation of the activation effect by cotransformation of *IbMYB340*, *IbbHLH2,* and *IbNAC56* on the *IbANS* promoter using a dual-luciferase assay in tobacco leaves. The ratio of Luc to Ren indicates that TFs activate the promoter activity of *IbANS*. **g** Y1H assays for *IbMYB340*, *IbNAC56a*, and *IbNAC56b* for the *IbANS* promoter. The prey vectors pGADT7-*IbMYB340*/*IbNAC56a*/*IbNAC56b* were transformed into Y1H Gold cells harboring pAbAi-*IbANS1/2/3* and then tested on SD/−Ura/−Leu/AbA^400^ plates

We speculated that the products of the *IbMYB340*, *IbbHLH2, IbNAC56a* and *IbNAC56b* genes might form regulatory complexes to regulate anthocyanin synthesis. As such, the possible interactions of *IbMYB340* with *IbbHLH2*, *IbNAC56a* and *IbNAC56b* were studied via Y2H assays. First, we found that cotransformed yeast cells harboring pGADT7 and *IbMYB340* failed to grow on SD − Trp/−Leu/−His/−Ade media, indicating that *IbMYB340* could not activate downstream gene expression in yeast by itself. As a result, strong growth on SD-Trp-Leu-His-Ade + AbA media was observed when we cotransformed the complete amino acid sequence of *IbMYB340* with *IbbHLH2, IbNAC56a* or *IbNAC56b*. However, the Y2H assays showed no interaction between *IbbHLH2* and *IbNAC56a* or *IbNAC56b* (Fig. 6a). These results suggest that *IbMYB340* can interact in yeast with *IbbHLH2*, *IbNAC56a* and *IbNAC56b*.

Additionally, we further validated the results obtained in yeast via firefly luciferase complementation assays in tobacco. Coexpression of NLuc-*IbMYB340* and CLuc-*IbbHLH2* or *IbNAC56a* or *IbNAC56b* vectors reversed the intense luciferase enzyme activity. In contrast, we detected no apparent luciferase enzyme activity in any of the control groups, including the groups with Nluc-*IbMYB340* with CLuc and CLuc-*IbbHLH2*/−*IbNAC56a*/−*IbNAC56b* with NLuc (Fig. 6b-d). Taken together, these results indicated that *IbMYB340* can interact with *IbNAC56a* and *IbNAC56b* in tobacco leaves. These results were in agreement with the results of the yeast two-hybrid assay.

We next tested the transactivation activity of the candidate TFs with the *IbANS* promoter via dual-luciferase reporter assays (Fig. 6f). Cotransformation of *IbMYB340*, *IbbHLH2* and *IbNAC56a* or *IbNAC56b* showed more transactivation on the *IbANS* promoter than did *IbMYB340* cotransformation with *IbbHLH2* and *IbNAC56a* or *IbNAC56b*. However, it seemed that cotransformation of *IbMYB340* and any other TFs or *IbMYB340* alone made no difference, while the transformation of *IbbHLH2* or *IbNAC56b* slightly promoted the activity (Fig. 6f). Furthermore, Y1H assays were performed to demonstrate whether the *IbANS* promoter region is bound directly by *IbMYB340* and *IbNAC56a* or *IbNAC56b.* Promoter structure analysis revealed multiple cis-regulatory elements, including a MYB motif (T/CAACCA) and a NAC-binding site (CACG) (Fig. 6e). In this assay, we transformed pGADT7-*IbMYB340/IbNAC56a/IbNAC56b* prey vectors into Y1H Gold cells harboring pAbAi-*IbANS1/2/3* bait vectors and tested them on SD/−Ura/−Leu/AbA plates. The transformants coexpressing the prey vectors pGADT7-*IbMYB340* and pAbAi-*IbANS1/3* were grown on SD/−Ura/−Leu/AbA^400^ plates, while the pAbAi-*IbANS1/2/3* bait vectors could not grow on SD/−Ura/AbA^400^ plates, suggesting that *IbMYB340* was capable of binding to the 1st (− 956 bp to − 755 bp) and 3rd (− 310 bp to − 105 bp) *IbANS* promoter fragments rather than the 2nd *IbANS* promoter fragment (Fig. 6g). However, we found no direct association between *IbNAC56a* or *IbNAC56b* and the promoter of *IbANS*, although several NAC-binding sites were located in different individual promoter regions (Fig. 6g). Taken together, these results indicated that the different regulatory complexes (*MYB340-bHLH2-NAC56a* and *MYB340-bHLH2-NAC56b*) directly activated the expression of *IbANS* by binding to the MYB motif element.

### *IbMYB44* suppresses anthocyanin accumulation by competitively inhibiting the regulatory complex formation of *IbMYB340-IbbHLH2-IbNAC56a* or *IbNAC56b*

**Fig. 7:**
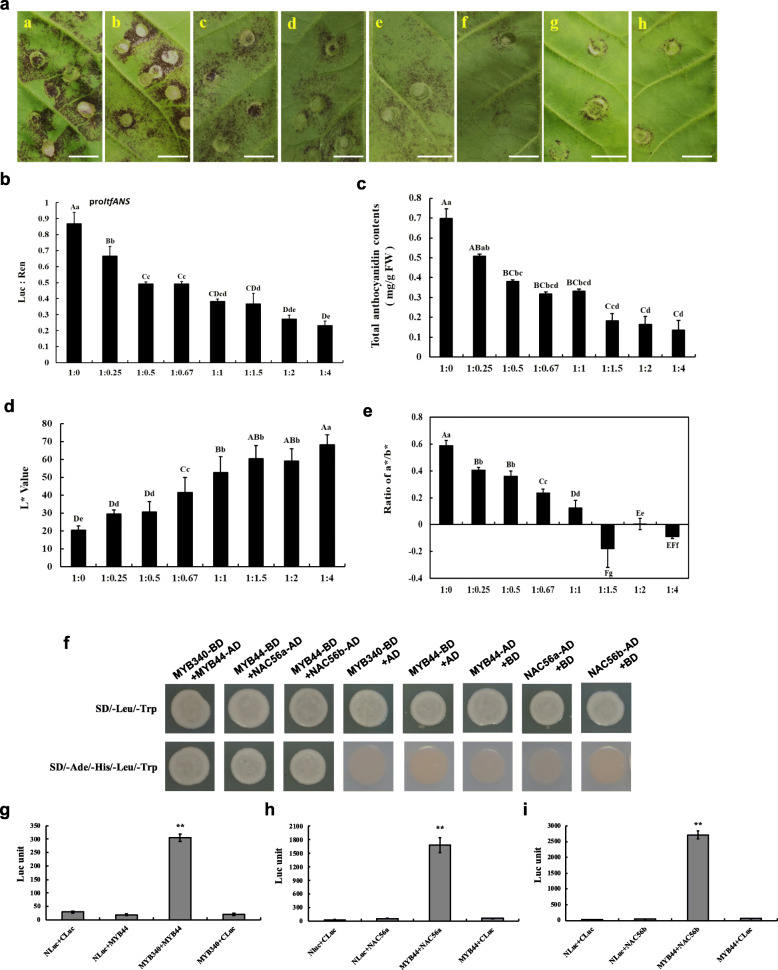
Functional analysis of the *IbMYB340* and *IbMYB44* genes using transient expression assays in tobacco leaves and interaction verification of *IbMYB44* with *IbMYB340*, *IbNAC56a* or *IbNAC56b* in vivo. **a** The phenotypes of tobacco leaves on the 7th day after infiltration; the letters ‘a’ ~ ‘h’ represent the ratio of Agrobacteria harboring the *IbMYB340* and *IbMYB44* constructs that were coinfiltrated into young tobacco leaves. Scale bar = 1 cm. **b** Transcriptional effects of coinfiltration of *IbMYB340* and *IbMYB44* at different ratios on *IbANS* promoter activity. **c** Anthocyanin contents in transformed leaves of tobacco. FW, fresh weight. **d**-**e** The color parameter L* and a*/b* ratio values were used to indicate phenotypic changes. The values are presented as the means ± SDs (n = 3), and the uppercase and lowercase letters represent significant differences in ratios at *P* < 0.01 or *P* < 0.05, respectively. **f** Verification of the interaction between *IbMYB44* and *IbMYB340*, *IbNAC56a*,or *IbNAC56b* via Y2H assays. **g** Verification of the interaction between *IbMYB340* and *IbMYB44* via firefly luciferase complementation assays. **h**-**i** Verification of the interaction between *IbMYB44* and *IbNAC56a* or *IbNAC56b* via firefly luciferase complementation assays

To study the role of *IbMYB44* in anthocyanin biosynthesis, we cotransformed *IbMYB340* and *IbMYB44* at different ratios into the abaxial side of tobacco leaves to test the transcriptional repression effect of *IbMYB44*. As shown in Fig. 7a, the pigmentation in tobacco leaves gradually diminished with an increasing proportion of *IbMYB44*. Using a dual-luciferase reporter assay, we then investigated the transactivation activity of the *IbANS* promoter when *IbMYB44* and *IbMYB340* were cotransformed in tobacco leaves. The results showed that steadily declining *IbANS* promoter activity occurred when the *IbMYB340*:*IbMYB44* ratio decreased from 1:0 to 1:4. However, it seemed that there were no changes between several ratios of *IbMYB340*:*IbMYB44*: 1:0.5, 1:0.67, 1:1 and 1:1.5 (Fig. 7b). The total anthocyanin contents and a*/b* values declined significantly at different ratios, their changes were consistent with the phenotypes of the tobacco leaves mentioned above, and the L* values apparently increased (Fig. 7c-e). Thus, when cotransformed with *IbMYB340*, *IbMYB44* coulddecrease anthocyanin biosynthesis.

We further verified the interaction of *IbMYB44* with *IbMYB340* and *IbNAC56a* or *IbNAC56b*. Y2H analysis showed that the cells cotransformed with *IbMYB44* with *IbMYB340* and *IbNAC56a* or *IbNAC56b* could grow on SD-Trp-Leu-His-Ade + AbA plates (Fig. 7f). Moreover, the marked luciferase enzyme activity was rescued by the control infiltrated with *IbMYB340* + *IbMYB44*, *IbMYB44* + *IbNAC56a* or *IbMYB44* + *IbNAC56b*; by contrast, Nluc-*IbMYB340/IbMYB44*coinfiltrated with CLuc or CLuc-*IbMYB44/IbNAC56a/IbNAC56b* coinfiltrated with Nluc did not result in a sufficient level of luciferase enzyme activity (Fig. 7g-i). Overall, *IbMYB44* could interact with *IbMYB340, IbNAC56a* or *IbNAC56b*, suggesting that *IbMYB44* suppressed anthocyanin accumulation probably through competitive inhibition of *IbMYB340, IbNAC56a* or *IbNAC56b*.

## Discussion

Anthocyanins have gained much attention in recent years and are common flavonoid compounds involved in red or purple pigmentation in plants [33]. To date, it is well known that structural genes participating in the anthocyanin pathway are regulated by the MYB-bHLH-WD40 complex in plants, and R2R3-MYBs particularly contribute greatly to anthocyanin accumulation; TFs have been extensively studied in different plant species, including *AtMYB114* in *Arabidopsis, PpMYB10.1* in peach, *PyMYB114* in pear, *MdMYB110a* in apple, and *IbMYB1* in sweet potato [8, 10, 13, 15, 30]. Moreover, coexpression of *PpMYB10.1* and *PpbHLH3/33* can promote anthocyanin biosynthesis in peach [9]. In addition, two activators of MYBs, *PyMYB114* and *PyMYB10*, can interact to promote anthocyanin biosynthesis in red pears [15]. In this study, the TF *IbMYB340*, which was highly homologous to *AtPAP1* in *Arabidopsis,* could induce high levels of anthocyanin pigmentation, especially when cotransformed with other TFs, in tobacco leaves and strawberry receptacles (Figs. 3a, 4a). We further demonstrated that *IbMYB340* interacts with *IbbHLH2*, *IbNAC56a* or *IbNAC56b* to regulate anthocyanin synthesis by forming regulatory complexes. However, we detected no interaction between *IbbHLH2* and *IbNAC56a* or *IbNAC56b* (Fig. 6a). These results revealed that *IbMYB340* makes great contributions to anthocyanin accumulation as a transcriptional activator. However, the relationship of *IbMYB1* [30] with *IbMYB340* and other TFs in regulating anthocyanin biosynthesis in purple-flesh sweet potatoes is unclear, and the synergistic regulation of color formation needs to be studied further.

MYB repressors have recently gradually been shown to participate in regulating the flavonoid biosynthetic pathway. The repressors usually contain an LxLxL negative repressor motif located in the C-terminal region, which is responsible for the repressive effect [22]. As reported recently, the enhanced expression of flesh-specific *StMYB44* accounts for the reduced contents of anthocyanin in potato flesh [22]. *CmMYB#7*, a negative R3-MYB regulator in chrysanthemum, can competitively inhibit *CmMYB6* for interaction with *CmbHLH2*, resulting in a decrease in anthocyanin content [20]. In peach, the *PpMYB18* protein can repress anthocyanin accumulation by competitively inhibiting the interaction of MYB activators with bHLHs [21]. Here, the R2R3-MYB *IbMYB44* caused the pigmentation to diminish gradually in tobacco in the injection regions (Fig. 7a). This was consistent with the results of a previous study in that the MYB repressors containing the LxLxL motif resulted in a decrease in anthocyanin biosynthesis. Then, we obtained evidence of the interactions between *IbMYB44* and *IbMYB340* and between *IbMYB44* with *IbNAC56a* or *IbNAC56b* by Y2H and firefly luciferase complementation assays (Fig. 7f-i). The results indicated that the *IbMYB44*-*IbMYB340, IbMYB44-IbNAC56a* or *IbMYB44-IbNAC56b* complexes might inhibit the formation of the regulatory complexes *MYB340*-*bHLH2*-*NAC56a* or *MYB340*-*bHLH2*-*NAC56b*. Taken together, the results show that *IbMYB44* can negatively regulate anthocyanin biosynthesis in sweet potato.

The MBW complex is popularly recognized as a regulator affecting anthocyanin biosynthesis at the transcriptional level. In addition to the MBW complex, numerous TFs, including WRKYs, NACs, ERFs, COP1, SPL9, DELLA proteins and so on, have been shown to corroborate in determining anthocyanin biosynthesis or indirectly affect the activity of the MBW complex [15, 27, 28, 34–37]. For the NAC TF family, it was reported that a NAC TF designated BLOOD (BL) acts as a heterodimer with *PpNAC1* to promote the transcriptional activity of *PpMYB10.1*, leading to anthocyanin accumulation [28]. Moreover, a negative effect of *ANAC032* on anthocyanin accumulation was investigated in *Arabidopsis* in response to stress conditions [24]. In this study, three NAC genes, *IbNAC56a*, *IbNAC56b,* and *IbNAC25*, were identified based on the results of a phylogenetic analysis of 98 *Ib*NACs and *PpBL* (Fig. 1a). RT-qPCR analysis showed that *IbNAC56a/IbNAC56b* were significantly expressed in ‘Xuzi No. 8’ and ‘Zhezi No. 3’ with abundant anthocyanin content (Fig. 2a), suggesting that they may function as positive regulators of anthocyanin biosynthesis in sweet potato tuberous roots. More obvious pigmentation was clearly detected when we cotransformed *IbNAC56a* or *IbNAC56b* with *IbMYB340* and *IbbHLH2* in heterologous overexpression systems (Figs. 3a, 4a), suggesting that *IbNAC56a* or *IbNAC56b* has an additive effect on the fully functional *MYB340*-*bHLH2* partnership. The regulatory pattern was similar to that reported in peach [28]. In addition, although *IbNAC25* is homologous to *PpBL*, the expression pattern was different from that of *IbNAC56a* or *IbNAC56b* in purple-flesh sweet potatoes, and the correlation analysis also showed that *IbNAC25* was negatively correlated with other factors except *IbMYB44*, *IbDFR* and *IbUFGT* (Fig. 2b). As such, we speculated that *IbNAC25* might negatively regulate anthocyanin biosynthesis; however, the function of this gene needs to be studied further.

As reported, the storage roots of ‘Yamakawamurasaki’, a purple-fleshed sweet potato cultivar, had a markedly higher expression level of *IbANS* than the white-fleshed cultivar ‘Yubeibai’, while *IbDFR* or *IbUFGT* showed no obvious differences [29]. In this study, we found similar results: the yellow−/white-fleshed cultivars showed lower *IbANS* expression than did the purple-fleshed cultivars, while the expression levels of *IbUFGT* and *IbDFR* had no obvious correlation in these cultivars (Fig. 2a). In this study, cotransformation of *IbMYB340*, *IbbHLH2* and *IbNAC56a* or *IbNAC56b* induced apparent anthocyanin accumulation in tobacco leaves and strawberry receptacles (Figs. 3a, 4a). In addition, RT-qPCR analysis showed that the expression of *FvANS* was highly upregulated with the cotransformation of the above mentioned TFs (Fig. 5a). The Y1H assay illustrated that *IbMYB340* could directly bind to the *IbANS* promoter, and the *IbANS* promoter activity was the strongest when *IbMYB340*, *IbbHLH2* and *IbNAC56a* or *IbNAC56b* were cotransformed, as reflected by a dual-luciferase reporter assay (Fig. 6b-c), suggesting that the *IbANS* gene may play a vital role in the regulatory network of anthocyanin biosynthesis in various sweet potato cultivars.

## Conclusions

In summary, using Y2H, firefly luciferase complementation, and dual-luciferase reporter assays, we investigated whether different regulatory complexes (*MYB340*-*bHLH2*-*NAC56a* or *MYB340*-*bHLH2*-*NAC56b*) can be formed to promote anthocyanin biosynthesis by binding to the *IbANS* promoter and upregulating the expression of other anthocyanin-related genes. In addition, our findings illustrated that *IbMYB44* acts as a repressor of anthocyanin biosynthesis in sweet potato by interacting with *IbMYB340* and *IbNAC56a* or *IbNAC56b* gene function or by indirectly affecting the *MYB340*-*bHLH2*-*NAC56a* or *MYB340*-*bHLH2*-*NAC56b* complex, which can enhance *IbANS* promoter activity. These results can help us develop a stronger understanding of the possible underlying mechanism of anthocyanin biosynthesis in purple-flesh sweet potato, although additional scientific research needs to be done to give a comprehensive and profound interpretation.

## Methods

### Plant materials

The storage roots of ‘Hanzi’, ‘Xuzi No. 8’, ‘Zhezi No. 3’, ‘Zhezi No. 4’, ‘Sushu No. 8’, ‘Guangshu No. 87’, ‘Lizixiang’, and ‘Xushu No. 18’ sweet potato were collected at 120 days after budding in the field at the National Sweet Potato Improvement Center (Xuzhou, Jiangsu Province, China) on 10 October 2018.

The seeds of tobacco (*Nicotiana tabacum* ‘NC89’ and *Nicotiana benthamiana*) are preserved and presented in School of Food and Biological Engineering, Hefei University of Technology (Hefei, Anhui Province, China), and the plants were grown in the glasshouse at 24 °C under artificial irradiance (daylight, 16 h). Four-week-old *Nicotiana tabacum* ‘NC89’ leaves were used for the transient transformation experiment and for the dual-luciferase reporter system assay, and tobacco (*Nicotiana benthamiana*) plants with six leaves were used for firefly luciferase complementation assays.

The seeds of ‘Yellow Wonder’ 5AF7 (YW5AF7) was provided by Prof. Jun Wu (Nanjing Agricultural University, China), which is a diploid strawberry (*F. vesca*) cultivar with yellow-white colored fruit. It was grown in the glasshouse under 12 h of light, while the temperature was maintained at 25 °C during the daytime and at 20 °C during the night. Receptacles 2 weeks after anthesis were used for transient expression assays.

### RNA extraction and qPCR analysis

The total RNA of 0.8 g of sweet potato tuberous roots and 0.5 g of strawberry samples was extracted using a Plant Total RNA Isolation Kit (Foregene). First-strand cDNA was synthesized from total RNA using Prime Script™ RT Master Mix (Takara). qRT-PCR was conducted using SYBR® Premix Ex Taq™ II (Takara) in a total reaction volume of 20 μl consisting of 150 ng of template cDNA, each primer at 0.2 μM and 10 μl of SYBR® Premix Ex Taq™ II, and the amplification program was as follows: 1 cycle of 95 °C for 10 s followed by 40 cycles of 95 °C for 5 s and 60 °C for 34 s. A strawberry gene, *FvTubulin* (gene11892), and a sweet potato gene, *IbTubulin* (*Itf*04g29110), were used as internal controls. The primers used for RT-qPCR are listed in additional file 1: Table S1.

### Transient assays in tobacco and strawberry

For the transient expression analysis, PCR amplification was conducted using Phanta® Super-Fidelity DNA Polymerase (Vazyme), and the full-length coding sequences of *IbMYB340* (*Itf*12g05820.t1), *IbbHLH2* (*Itf*14g18730.t2), *IbNAC56a* (*Itf*02g15460.t1) and *IbNAC56b* (*Itf*01g19290.t1) were inserted into a pSAK277 vector under the control of the 35S promoter with *EcoR*I and *Xba*I. The primer sequences used for expression vector construction are listed in Additional file 1: Table S2. The recombinant vectors were individually transformed into GV3101 strains of *Agrobacterium tumefaciens* using the chemical method. The Agrobacterium cells harboring the recombinant pSAK277 vectors was resuspended in an injection solution (OD_600_ = 1.0) and incubated at 25 °C under 60 rpm at 4–5 h before injection.

For the functional assay of *IbMYB340*, *IbbHLH2* and *IbNAC56*, Agrobacterium cultures containing the abovementioned TFs were mixed equally. In addition, Agrobacterium cells harboring *IbMYB340* and *IbMYB44* were mixed at different ratios to test the repressing effect of *IbMYB44*. The mixed Agrobacterium cells were injected into young *N. tabacum* leaves and strawberry receptacles according to the methods described by Zhou et al. [21]. The plants were incubated in darkness for 24 h and then moved to a greenhouse with artificial irradiance (16 h daylength). Photos were taken 7 d after injection, anthocyanins were collected for quantification, and total RNA was collected for extraction where necessary.

### Extraction and quantification of anthocyanins in tobacco and strawberry

Anthocyanins were extracted and quantified according to the methods described by Yao et al. [15]. In brief, we immersed 0.2 g of tobacco leaf tissue or 0.2 g of strawberry receptacle tissue around the injection regions, which had been fully groundin liquid nitrogen, in 1 ml of cold methanol consisting of 0.1% HCl at 4 °C for 24 h. The supernatant was collected after centrifugation of the mixture at 12000 g for 15 min. The anthocyanin levels were estimated from the methanolic extracts according to the eq. A = (A_530_ - A_620_) – 0.1 (A_650_ - A_620_), and the absorbance was measured using a Multiskan Spectrum device (Thermo Scientific Multiskan GO 1510, Finland).

### Yeast one-hybrid assay

A yeast one-hybrid (Y1H) assay was performed using a Matchmaker® Gold Yeast One-Hybrid System. Briefly, three amplifications of *IbANS* promoter fragments (− 956 bp to − 755 bp, − 396 bp to − 224 bp and − 310 bp to − 105 bp) were cloned into a pAbAi vector with *Hind* III and *Xho* I, and the complete sequences of *IbMYB340*, *IbNAC56a* and *IbNAC56b* were cloned into a pGADT7 vector with *EcoR* I and *Xho* I. Then, we transformed prey vectors into Y1H Gold cells harboring the pAbAi-bait and tested them on SD/−Ura/−Leu/AbA plates. The primer sequences used for vector construction are listed in Additional file 1: Table S2.

### Dual-luciferase reporter assay of tobacco leaves

To construct the dual-luciferase reporter vector, the 1.9 kb upstream promoter region of *IbANS* (*Itf*13g04110.t1) (from the ATG start codon) was amplified from the genomic DNA of tuberous roots from the sweet potato cultivar ‘Xuzi No. 8’ and inserted into a pGreen II 0800-LUC binary vector. Moreover, Agrobacterium transformation and injection preparation were the same as the methods described for the transient transformation assay in tobacco leaves and strawberries.

Agrobacterium cells harboring the pGreen II 0800-LUC recombinant vector, pSAK277 vector, *IbMYB340*, *IbbHLH2,* and *IbNAC56* were mixed at a 1:3:3:3 ratio. The mixture of Agrobacterium cells was injected into young *N. tabacum* leaves that were 2 weeks old. At 48–72 h after infiltration, the LUC and Ren activity were measured with an E1910 Dual-Luciferase® Reporter Assay System (Promega).

### Yeast two-hybrid assay

A yeast two-hybrid (Y2H) assay was performed using a Matchmaker® Gold Yeast Two-Hybrid System. The full-length coding sequences of *IbMYB340*, *IbbHLH2* and *IbMYB44* were amplified and cloned into a pGBKT7 vector with *Nde*I and *Pst*I; moreover, the complete sequences of *IbbHLH2*, *IbNAC56a*, *IbNAC56b* and *IbMYB44* were cloned into pGADT7 with *EcoR*I and *Xho*I. Then, we cotransformed the integrated vectors into Y2H Gold cells with the LiCl-PEG method according to the manufacturer’s instructions and tested the protein-protein interactions on SD/−Leu/−Trp/−His/−Ade + AbA plates.

### Firefly luciferase complementation assay

The firefly luciferase complementation assay was performed according to the methods reported by Chen et al. [38]. Full-length coding sequences of *IbMYB340* and *IbMYB44* without stop codons were cloned into a pCambia1300-NLuc binary vector, and the complete sequences of *IbNAC56* and *IbMYB44* were cloned into a pCambia1300-CLuc binary vector. Agrobacterial transformation and injection preparation were performed as previously described for the transient transformation assay. Then, the activity of firefly luciferase was measured with a Steady-Glo® Luciferase Assay System (Promega) during 48–72 h.

### Statistical analysis

All samples were replicated at least three times independently, and all the data are represented as the means ±SDs. Statistical analyses were performed using Student’s *t*-test embedded in Excel 2013 software. Pearson’s correlation coefficients (R values) and the constructed heatmap were analyzed using R scripts. Significance is indicated by * (*P* < 0.05) or ** (*P* < 0.01) or by different letters.

## Supplementary information

**Additional file 1: Figure S1.** Amino acid sequence alignment of *IbNACs* and the NAC transcription factor *PpBL* (ALK27819.1) in peach (*Prunus persica*). **Table S1.** List of primers used for RT-qPCR. **Table S2.** The list of primers used for developing the constructs. **Table S3.** Protein sequences subjected to phylogenetic analysis and multiple sequence alignments.

## Data Availability

The datasets used and/or analysed during the current study available from the corresponding author on reasonable request.

## Abbreviations

TF: Transcription factor
PAL: Phenylalanine ammonia lyase
F3H: Flavanone 3-hydroxylase
DFR: Dihydroflavonol 4-reductase
ANS: Anthocyanidin synthase
UFGT: UDP-glucose flavonoid 3-O-glucosyltransferase
GST: Glutathione S-transferase
Y2H: Yeast two-hybrid
Y1H: Yeast one-hybrid
MBW: MYB-bHLH-WD40
