# Supplementary material for: MYB44 competitively inhibits the formation of the MYB340-bHLH2-NAC56 complex to regulate anthocyanin biosynthesis in purple-fleshed sweet potato
Source: BMC Plant Biol. 2020 Jun 5;20:258. doi: 10.1186/s12870-020-02451-y (PMC7275474; doi:10.1186/s12870-020-02451-y)
Supplement: Supplementary file 1 — Additional file 1: Figure S1. Amino acid sequence alignment of IbNACs and the NAC transcription factor PpBL (ALK27819.1) in peach (Prunus persica). Table S1. List of primers used for RT-qPCR. Table S2. The list of primers used for developing the constructs. Table S3. Protein sequences subjected to phylogenetic analysis and multiple sequence alignments. [file 12870_2020_2451_MOESM1_ESM.pdf]

Additional file 1:

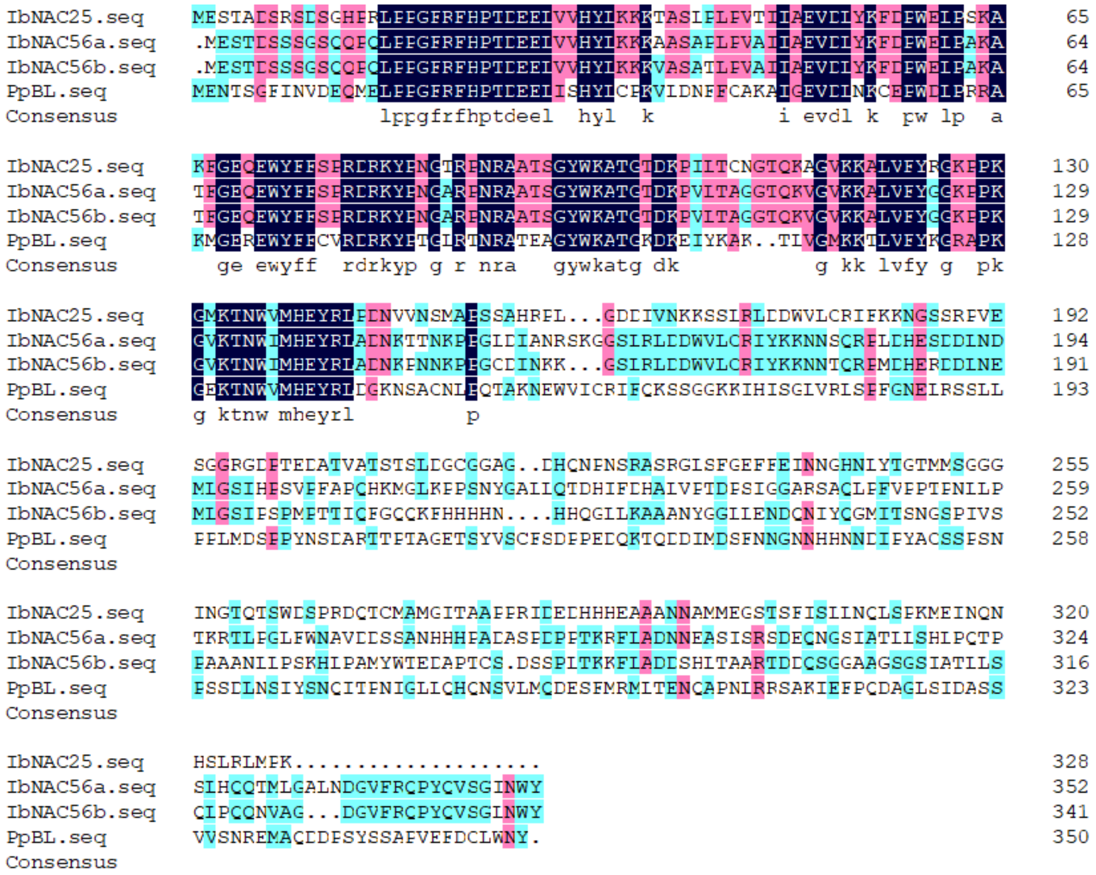

Figure S1. Amino acid sequence alignment of *IbNACs* and a NAC transcription factor *PpBL* (ALK27819.1) in peach (*Prunus persica*)

Table S1. The list of primers used for RT-qPCR

Gene name	Gene ID	Direction	Primer sequence (5'-3')
Ibtubulin	itf04g29110.t1	Forward	CTCAAGAGGGTCTCAGCAATAC
		Reverse	TGTCAAGTAACGCCCATGTC
IbNAC56a	itf02g15460.t1	Forward	CGCCAATAGATCAAAAGGCGG
		Reverse	GTCCCATCTTGTGCTGGGGA
IbNAC56b	itf01g19290.t1	Forward	TGCCCACAACAATCCAATTTGG
		Reverse	GTCCAGTACATGGCGGGGAG
IbNAC25	itf10g20230.t1	Forward	CCGGCGATCACCAAAATCCC
		Reverse	CTCTCGGTGAATCCCAGGAGG
IbMYB340	itf12g05820.t1	Forward	CCCGGAAGAACAGACAATGA
		Reverse	TTCTGCGGTTCTTTCTCCTATC
IbMYB44	itf03g30290.t1	Forward	GGACATGATAAAGACGGAGGTG
		Reverse	GGTGGTGGTGTTGGTGATAA
IbbHLH2	itf14g18730.t2	Forward	CCTACAAGTGAAGAGCGGTATG
		Reverse	CTGTAATCTCCCGAAGGACAAG
IbANS	itf13g04110.t1	Forward	TCAGCCAATTGAGGAGAAAGAG
		Reverse	CAAGCTGCCCACTAGCATTA
IbUFGT	itf03g22690.t1	Forward	CGGAACCAAGGACCAAGTAAA
		Reverse	GCTAAATCCGGGACCCAATAA
IbDFR	itf12g03120.t1	Forward	CAGTATGTGCACCTGGATGAT
		Reverse	TGGATGGTTGTATGGTGAGAAG
Housekeeping	gene11892	Forward	AGCCTAACGCAGAGGTTCCAAA
		Reverse	GCAGCCCACATTGAAGGGTCTATAGT
FvANS	gene32347	Forward	GAAGTGCGTACCCAACTCCATCGT
		Reverse	ACCTTCTCCTTGTTGACGAGCCC
FvDFR1	gene15174	Forward	CACGATTCACGACATTGCGAAATT
		Reverse	GAACTCAAACCCCATCTCTTTCAGCTT
FvUFGT	gene12591	Forward	TTTGGTTCGGTGCTCATA
		Reverse	AGCATCTGTCCCATCTGGT
FvRAP	gene31672	Forward	CAAGTTCCAGCAATCGAAGA
		Reverse	TGGGAAGGATCACAAGTTGA
FvF3H	gene25801	Forward	TGTCCATAGCGACATTCCAGAACC
		Reverse	CTCTGAATCCTGCAGTTGCTCCTT
FvPAL2	gene23261	Forward	TGGCGCTTAACGGAAACGGAAA
		Reverse	GGCTTCCGGTAGTCATTCACCATT

Table S2. The list of primers used for making constructs

Construct	Primer name	Primer sequence (5'-3')
PSAK277-IbMYB340	277-MYB340-F	ACTAGTGGATCCAAAGAATTCATGGTGGGAGCTGCTGAGAA
	277-MYB340-R	TCATTAAAGCAGGACTCTAGATCAGAAAATAAACCCTCCATTCCA
PSAK277-IbbHLH2	277-b2-F	ACTAGTGGATCCAAAGAATTCATGGCGGCGGAAAACCCT
	277-b2-R	TCATTAAAGCAGGACTCTAGACTAACCGGAGGTGTTCTTCCTTG
PSAK277-IbNAC56a	277-NAC56a-F	ACTAGTGGATCCAAAGAATTCATGGAGAGCACCGATTCATCG
	277-NAC56a-R	TCATTAAAGCAGGACTCTAGATTAAGAGTACCAATTGATGCCGG
PSAK277-IbNAC56b	277-NAC56b-F	ACTAGTGGATCCAAAGAATTCATGGAGAGCACCGATTCGTCG
	277-NAC56b-R	TCATTAAAGCAGGACTCTAGATCAAGAGTACCAATTGAGGCCG
PSAK277-IbMYB44	277-itf290-F	ACTAGTGGATCCAAAGAATTCATGGCGAGTATTAGTCCTAATGGG
	277-itf290-R	TCATTAAAGCAGGACTCTAGACTACTCTAATCGGTTAACTCCGACG
0800-IbANS	0800-ANS-F	GTCGACGGTATCGATAAGCTTGGAGGTCGTTCGTTCGAAAA
	0800-ANS-R	CGCTCTAGAACTAGTGGATCCATTTTCATTCGATCTTGATTGTGTAGA
BK-MYB340	BK-MYB340-F1	TCAGAGGAGGACCTGCATATGATGGTGGGAGCTGCTGAGAA
	BK-MYB340-R4	CTAGTTATGCGGCCGCTGCAGGAAAATAAACCCTCCATTCCAGAG
BK-MYB44	BK-MYB44-F	TCAGAGGAGGACCTGCATATGATGGCGAGTATTAGTCCTAATG
	BK-MYB44-R	CTAGTTATGCGGCCGCTGCAGCTCTAATCGGTTAACTCCGAC
BK-IbbHLH2	BK-b2-F	TCAGAGGAGGACCTGCATATGATGGCGGCGGAAAACCCT
	BK-b2-R	CTAGTTATGCGGCCGCTGCAGCTAACCGGAGGTGTTCTTCCTTG
AD-IbbHLH2	AD-b2-F	GCCATGGAGGCCAGTGAATTCATGGCGGCGGAAAACCCT
	AD-b2-R	ACGATTCATCTGCAGCTCGAGCTAACCGGAGGTGTTCTTCCTTG
AD-NAC56a	AD-NAC56a-F	GCCATGGAGGCCAGTGAATTCATGGAGAGCACCGATTCATCG
	AD-NAC56a-R	ACGATTCATCTGCAGCTCGAGAGAGTACCAATTGATGCCGGAA
AD-NAC56b	AD-NAC56b-F	GCCATGGAGGCCAGTGAATTCATGGAGAGCACCGATTCGTCG
	AD-NAC56b-R	ACGATTCATCTGCAGCTCGAGAGAGTACCAATTGAGGCCGGAG
AD-MYB44	AD-MYB44-F	GCCATGGAGGCCAGTGAATTCATGGCGAGTATTAGTCCTAATGGG
	AD-MYB44-R	ACGATTCATCTGCAGCTCGAGCTCTAATCGGTTAACTCCGACGCGA
Nluc-MYB44	Nluc-MYB44-F	CGAGCTCGGTACCCGGGATCCATGGCGAGTATTAGTCCTAATGGG
	Nluc-MYB44-R	CGCGTACGAGATCTGGTCGACCTCTAATCGGTTAACTCCGACGCGA
Nluc-MYB340	Nluc-MYB340-F	CGAGCTCGGTACCCGGGATCCATGGTGGGAGCTGCTGAGAAAG
	Nluc-MYB340-R	CGCGTACGAGATCTGGTCGACGAAAATAAACCCTCCATTCCAGAG
Cluc-NAC56a	Cluc-NAC56a-F	CCGGGGCGGTACCCGGGATCCATGGAGAGCACCGATTCATCG
	Cluc-NAC56a-R	ACGAAAGCTCTGCAGGTCGACTTAAGAGTACCAATTGATGCCG
Cluc-NAC56b	Cluc-NAC56b-F	CCGGGGCGGTACCCGGGATCCATGGAGAGCACCGATTCGTCG
	Cluc-NAC56b-R	ACGAAAGCTCTGCAGGTCGACTCAAGAGTACCAATTGAGGCCG
Cluc-MYB44	Cluc-itf290-F	CCGGGGCGGTACCCGGGATCCATGGCGAGTATTAGTCCTAATGGG
	Cluc-itf290-R	ACGAAAGCTCTGCAGGTCGACCTACTCTAATCGGTTAACTCCGACG
pAbANS1	pAbANS1-F	AAATGATGAATTGAAAAGCTTCTTAACATTACATCACGAAAGTTGTCC
	pAbANS1-R	ATACAGAGCACATGCCTCGAGGAGTGTTTGCTTAACTGCATGTGA
pAbANS2	pAbANS2-F	AAATGATGAATTGAAAAGCTTACTCTAGAGAATTCTTGAGATGCAAACG
	pAbANS2-R	ATACAGAGCACATGCCTCGAGGTATATATCCCACCGGATGTACAACG
pAbANS3	pAbANS3-F	AAATGATGAATTGAAAAGCTTGTAAATATAATAATAGTTTTCCTCCGGC
	pAbANS3-R	ATACAGAGCACATGCCTCGAGGGTGAGCACGTGATGATGAGATG

AD-MYB340	AD-MYB340-F	GCCATGGAGGCCAGTGAATTCATGGTGGGAGCTGCTGAGAA
	AD-MYB340-R	ACGATTCATCTGCAGCTCGAGGAAAATAAACCCTCCATTCCAGAG

Table S3. Protein sequences for phylogenetic analysis and multiple sequence alignments

NACs

>itf00g28880.t1.
MEDMPVGFRFYPTEEELVAFYLGNKLRGERPDIDLVIPVVNIYLHRPWELPQLAGV
ASYGDDEQWFYFMPGPENIARGGKPNRLTTEGYWKATGCPSLVFSINNRIIGEKRT
MVFYTGRAPNGTKTEWKINEYKAVHGEASASAPLSDLEVSCRHKSIGDWALGAK
YL*
>itf00g28890.t1.
MEDLPTGFRFYPTEEELVSFYLRNKLRGERPDIDLVIPVVNIYLHRPWELPQLAGVV
GGHGDYDQWFYFVPGPENMARGGKPNRLTTEGYWKATGSPGLVFSLNNRVIGEK
RTMVFYTGRAQNGTKTEWKINEYKAVRGDASASTPLSNLELCQEFSLCRLYKKSK
CDRAFDRRPLDLTATRRRIAQLPPQDNVQTTPITVQEPSATESTGAGKISPPGAAVIP
SNNATATPLYWDNFEPIWDWEDCFSLL*
>itf00g38350.t1.
MEKQKAAALTGRGNLDLALAAPTRRLGPGFRFHPTDEELVVYYLRRKVRRKPFH
VEAIAVVDIYKHEPWELHAFSAVNSTDQEWYFLVDLEKKYKGSSRLINRSFTEKG
YWKTSEKDQTVSHKREIVGMKKTLVYHAGKPPNGRRTNWVMHEYRLVEAELDK
AGVQQVHRDLLLHYPAHPAAC*
>itf00g48300.t1.
MRLNFLFRFVLSVFEQRLKMMRLNFCVADMALYGEKEWYFFSPRDRKYPNGSRP
NRAAGRGYWKATGADKPIGQPKPVGIKKALVFYSGKAPKGEKTNWIMHEYRLAD
VDRSARKKSNSLRLDDWVLCRIYNKKGELEKADVSIRNMSKATASSPVSEEEEKK
PEMMLPPPPQPQPPQVFSDFLYLEPSDSIPKLHTESSCSEHVLSPEFTCDREVQSEAK
MSEWDKTALNFPFNYQNATADGGFLGCGDLPGSYDAWPLQDMLLYKPF*
>itf01g00340.t1.
MLGIEEILCELNRDEDNEQGLPPGFRFHPTDEELITFYLASKVFHGTFSGIHIVEVDL
NRCEPWELPDVAKMGEREWYFFSLRDRKYPTGLRTNRATGAGYWKATGKDREV
YSASSGALLGMKKTLVFYRGRAPRGEKTKWVMHEYRLDGHFSCRHTCKEEWVIC
RIYHKIGEKRNAIMVQAAGGGQSYPTLKTLTSSANVALPPFPEMQSQNPMQHHHH
HHKSIVFHHQENDLKSINHHHPLLPQTNLLGLPSINGVLSSSFSAITSNKTTPTTSKA
NTDKYNMLDCNNTILLPSSSSSSSILFKSQTGSSKSNAIATTAANCKISPCHLSETEK
YQNNPLFYGMGMGASPVTGFGFETMMPSAAATFDGMSTSPEGFTRTCSQMVDPPI
QLTADSWPMDL*
>itf01g19290.t1
MESTDSSSGSQQPQLPPGFRFHPTDEELVVHYLKKKVASATLPVAIIAEVDLYKFDP
WELPAKATFGEQEWYFFSPRDRKYPNGARPNRAATSGYWKATGTDKPVLTAGGT
QKVGVKKALVFYGGKPPKGVKTNWIMHEYRLADNKPNNKPPGCDINKKGSLRLD
DWVLCRIYKKNNTQRPMDHERDDLNEMLGSIPSPMPTTIQFGQQKFHHHHNHHQ
GLLKAAANYGGLLENDQNIYQGMITSNGSPIVSPAAANLLPSKHLPAMYWTEDAP
TCSDSSPLTKKFLADDSHLTAARTDDQSGGAAGSGSIATLLSQLPQQNVAGDGVFR
QPYQVSGLNWYS*

>itf01g30800.t1.
MELTSEPSSCSCLCGQRFPPGFRFQPTDEELAVYYLKRKICRRPIMLDVIGETDVYK
REPEELPELSKLKTGDRQWFFFSPRDRRYPNGARSNRATKQGFWKVTGRDRIITCN
SRNVGVKKTLVFYNKGRTPNIKPTRTDWVMYEYTMDEEELKRCPSAQDYYALYR
IFRKSGIGPKNGEQYGAPFREEDWADDDCLTVNPLVEQEKYTNQVNNVVNVESPIP
NDFEISSVNIDELLDLLEDEPCTLEPLVFGEDTESTLLDHSSQNPNLPAQNAILHPTT
PHNVQANFDVTQCSTLQPQLPEAPEFTSAPVYHGLNPQMVEEDYVEDFLEVDDLA
GPEPSINNFIKPGETMGIQQYGYDMGTGEARHPNQPYVNNAENGMVNPVSTYFSN
NHETMNSQQLYLNEDNEGSNRLWSPDQRCNVATPAEANHEVFHPATLDGWYGTT
MSSSKWRELFCPSGNGGV*
>itf01g30810.t1.
MVMEVKITSNSSSSASLSDQRFPPGFRFHPTDEELVLYYLKRKICHKKLLLDAICET
DVYKWDPEDLPELSKLKTGDRQWFFFSPRDRKYPNGARSNRATKHGYWKATGK
DRSITCNSRAVGVKKTLVFYKGRAPTGIRTDWVMHEYTLDEEEFRRCDSARDYYA
LYKVFKKSGAGPKNGEQYGAPFKEEEWDDDEYLDPNCSVDQKKSTNPVNDIQPTE
NPKPNVPFQYPKDDLEGFLNHIGNEPPPLMQPLSIDYSYDMEHLIGEEDSESTLLDQ
SSRELNVTEQTALQQYYMQASFEVTESGTSQPLLHEASEVTSSAVFNEQTQAVEED
FLEDFLEMDDLLSTEPDIHKFNMPVEKSDTQLLNDFDGLSEFDLYQDAAMFFHDV
GTSEGGQAAESYVNNVLNGTVNQASTYFSDYPEIVNGQQLYFNESNEVSDQLWM
HDQSSDIFNPDDINQGGIPSGVGYDNNFMNHPAGANPIQIARQDDATNSSLSSSLW
ALVESIPTTPASAAESALVNKAFERMSSFRRLRLKAINMNVAAGATSRSASKSRRGI
LCFSLLGVLCAILWVFIGTSFCVMGRCVSS*
>itf02g00760.t1.
MEENLPPGFRFHPTDEELITYYLGNKVSDFGFSPIAIADVDLNKCEPWDLPGKASM
GEKEWYFFSLRDRKYPTGLRTNRATEAGYWKTTGKDKEIFRGGVQLVGMKKTLV
FYRGRAPKGEKTNWVMHEYRLESKHGFKPTKEEWVVCRVFQKCNTIMKKPQATS
ASSPQSLDESPCDTNTTIANELGDIELPNFNTTIMPSPSNGIINNISLQTYNNNNNEG
MMNMNNLNMNNTLPLLSWPPSTSLLSSNLSSVNSLLFRALQLRGQGHHHQQAAIS
TSDYTGGYNNMPQFGNDLINSNDFTPTSTSSMVLDSANHHHHQQQQNPQDSHIW*
>itf02g03850.t1.
MVMEKLCEGVGDQMELPPGFRFHPTDEELITHYLSNKVLDSTFSAIAIAEVDMNK
VEPWDLPWKARIGEKEWYFFCVRDKKYPTGLRTNRATVAGYWKATGKDKEIFRG
KCLVGMKKTLVFYMGRAPKGVKTNWVSHEYRLEGQLSLQNLNKPSKVQNDWVL
CRVFQKSSGGKKVHISGMIRSNNYPAGNGPQNSPLLPPLSDSPPCHGGAAKANSYV
HCFSSKQQDMNMNMNMNLINFSPLPISPIIPNNPFTTNPLQIPLLPMQDPTTLRNWL
ANYGFKTEKEIASGVSQETGLSTDINTEISSAGKRCFQDPPSTPPPGPQDFDCLWSY*
>itf02g06750.t1.
MMEQQPAQEGGALVVASPAPPKRTLAPPTSLAPGFRFHPTDEELVRYYLRRRACG
KPFRFQAVSEIDVYKSEPWELAEYSYLKTRDLEWYFFSPVDRKYGNGSRLNRATG
KGYWKATGKDRPVRHKSQTVGMKKTLVFHCGRAPDGKRTNWVMHEYRLTDNE
LVEAGVTQDAFVLCRIFQKSALGPPNGDRYAPFVEEEWDDGKALVVPGGVAEDD
VANGDDARVDGNDLDQGALCKAPESPVEPLSLSFVCKRERSEEDPEPLSLAQSKRS
KQDCPSSSHANGSEDSTTTSQDPPTMMMTTNDSSPVLLEFPLLDPTEPRENQPTNA

PTFDSSTLEKSVPPGYLKFISNLENEILNVSMERETLKIEVMRAQAMINILQSRIDLL
NKENEDLRRVVRGG*
>itf02g06760.t1.
MESRGSSQLLAPGFRFHPTDEELVFYYLRRKVCAKPLRFDAISEIDIYKVEPWDLPG
MSRLKTRDLEWYFFSMLDKKYGNGSRTNRATEKGYWKTTGKDRAVNHRSKVVG
MKKTLVYHSGRAPRGQRSNWVMHEYRLVDEDLSKAGIVQDAFVLCRVFQKSGA
GPKNGEQYGAPFVEEEWEDDEIAMVPKADLPEEAELADDIFLDGDDLEQILGADIP
LDGALVPVNNTTGNIEDAAAHVEDFENILVDNVEYPCGLQHPDEPKPFDQPIHDFD
PKPVKREYMGESSNSMTSENVNCLLNEPLMDASDGFQFNDGTFLETNDLSNPVEA
NTSGFDMFDFLNFYDAKDDFQDVLFDSDIFIGNDEFLEGQSPFADKDVCNTTEQAV
LPNEKSVDAEHKNDIASSSKPEPTKLGSDFQHPFIKQASQMLGNIPAPPAFASEFPK
DAALRLNAATQASSSVHVTAGLIQISDLTLGSKHGNYNIILSFDFLQGAQSPASLEP
VGHGKMLSGGWLYFLLFWVLILSISFKVGTFIYAR*
>itf02g10140.t1.
MEAKKMVVAVAAAAAVEKDEDEEVILPGFRFHPTDEELVGFYLRRRVENKRISIE
LIKQVDIYKHDPWDLPKIGTHVGDKEWYFFCIRGRKYRNSVRPNRVTGSGFWKAT
GIDKPVYSTGGETHHCIGLKKSLVYYRGSAGKGTKTDWMMHEFRLPPKLADKNN
NICQEAEVWTLCRIFKRTTNYKRCMPDWRQQTSRQSTIVENNASSKNEAIMGAAQ
QEQRNQFYASNDQISMALSSYSSSSNTTTFWNSPKLLDNNHFFIGENPHWDELKSV
VDLATDPQSFCYSHT*
>itf02g12660.t1.
MTCSSDSGEDRPARLFAQNSGGNNNSVTINEITRRSGMRACPSCGHPIKCEQKAGI
HNLPGLPAGVKFDPSDQEILEHLEAKVSLDSHKLHPLIDEFIHTLEGEDGICYTHPEK
LPGVTKDGLVRHFFHRPSKAYTTGTRKRRKVHTDVDGNETRWHKTGKTRPVAAK
GMVKGYKKILVLYTNYGKQRKPEKTNWVMHQYHLGDDEDEKEGELVVSKVFFQ
TQPRQCGSLLRDSPPLDLPQAGRKAGSHLRESAATMVESFNPTLISFDQFDHNRPTS
RLLPSFNMHDTSFIP*
>itf02g13580.t1.
MENLPLGFRFYPTEEELVSFYLRRKLQGATSADIDLVIPVVNIYHHSPCHLPQVAGE
LSRGDSEQWFYFIPGEENRARGGKPNRLTRDGYWKATGSPSFVYSSMNEIIGEKRT
MVFHTGRAPGGTRTQWKMNEYKAAAQVVGGQGGASASCDAIADLKLCHEFSLC
RLYKKSKCDRSFDRRPPEMAAATGAGGGAPPPPPQHNHLPAALMERSSSSTADDY
ATPPSAMEYQYYWDTCAPLWDWEDLNINLL*
>itf02g15080.t1.
MDAFISYQQQRFDCGGGDANLPPGFRFHPTDEELITYYLLKKVVDSTFSARAIAEV
DLNKCEPWELPEKARMGEKEWYFFSLRDRKYPTGLRTNRATEAGYWKATGKDR
EIYSSKTCALVGMKKTLVFYRGRAPKGEKTNWVMHEYRLDGKFAYHYFSRASKD
EWVISRVFQKSGAAAAGGGKRKMTSINLYPEVSSPSSVSLPPLVESSSYNNTATSAL
AIENESCSYEGVHNAATKEHVPCFSTAAPQTFNPNASLFDLPAPPLTAAPNFSPLIDP
SSAASSTRFARNNNVGVPAFPSLRSLQENLHLPFFFPAATPPPMHGGLGDQMGGYG
SAIPGWAPATESQKVGSSELDCMWSY*
>itf02g15450.t1
MGVKDMDPLSQLSLPPGFRFYPTDEELLVQYLCRKVAGHDFSLQIIGDIDLYKFDP
WDLPSRANFGEKEWYFFSPRDRKYPNGSRPNRVAGTGYWKATGTDKIITTEGRKV

GIKKALVFYVGKAPKGTKTNWIMHEYRLCEAPRKTGSARLDEWVLCRIYKKNSSA
AAAAAQKPVSGVQSKDYSHGSSSSSSSQFDDMLESLPEINDQYFSLPRINSLKNLNL
QQDDKSSILRLNSGSFDWATLAGINSIPELGPGNQIPQSGHLNHANPADIYGGHSM
ALSFPVDDEAQSGIRVENNSGIYQPQNPNAFTHNFSNLLDPYGIRYANQPGSLAFRQ
Q*
>itf02g15460.t1.
MESTDSSSGSQQPQLPPGFRFHPTDEELVVHYLKKKAASAPLPVAIIAEVDLYKFDP
WELPAKATFGEQEWYFFSPRDRKYPNGARPNRAATSGYWKATGTDKPVLTAGGT
QKVGVKKALVFYGGKPPKGVKTNWIMHEYRLADNKTTNKPPGLDIANRSKGGSL
RLDDWVLCRIYKKNNSQRPLDHESDDLNDMLGSIHPSVPFAPQHKMGLKPPSNYG
ALLQTDHIFDHALVPTDPSIGGARSAQLPFVPPTPNLLPTKRTLPGLFWNAVDDSSA
NHHHPADASPDPPTKRFLADNNEASISRSDEQNGSIATLLSHLPQTPSLHQQTMLG
ALNDGVFRQPYQVSGINWYS*
>itf03g00520.t1
MTALYMGRSWLINSRGLAQKVRNASSPAENRIKDCGATRECPNCHHLIDNSDVSP
EWPGFPAGVKFEPSDVELLEHLAAKCGEGNSEPHMFVDEFIPTLEGDEGICYTHPE
NLPGVKKDGSSVHFFYRTKKAYATGTRKRRKISNEKGLVMEHVRWHKTGKTKAV
MENGVQKGCKKVMVLYRTTKKGEKPGKTNWVMHQYHLGIDEDEKEGGYVVSKI
YYQQQKQTDNAVLLSGESDMYANQAVPMTPMIAIPNPPRAGETPSCDDYNAALSP
AEEVEGGNKEQYVSLFSSDVEVKCEEYAGGCLAGESQAVDATDIDDFLLCDEIVG
SFDFDNLGSHHATSANINPMAQEANNTTSTNIADLVNLETEFSLQDLPFGSQDSISS
WLDRI*
>itf03g01630.t1.
MENPCVFDDEMNLPPGFRFHPTDEELITHYLSPKSLNAAFSAVAIGEVDLNRVEPW
DLPWRARMGEKEWYFFCVRDRKYPTGMRTNRATESGYWKATGKDKEIFTAKTL
VGMKKTLVFYSGRAPRGERTNWVMHEYRLEGHHQTFQTEWVICRIFKKTAGGKK
IHISALIRGGGDCSNNSTPSSLPPLTDLSQTTYFSDSADDQKPIMMASSFASSPSPSKN
SDVSPAPLIFPADPTPATYFHSDQIIPYADNMDFSDSGMTQDHSVPRFLHPYEEQAF
LMTSAAAGPVDIGCLWDY*
>itf03g04990.t1.
MEAMATVSLPPGFRFHPTDEELVAYYLKRKINGHRIELDVIPEVDLYKCEPWDLPG
KALLPSKDLEWYFFSPRDRKYPNGSRTNRATKAGYWKATGKDRKVNSEMRAVG
MKKTLVYHRGRAPHGARTDWIMHEYRLDERECETRSGLQEAYALCRIFKKSLNG
LKIGENSGADRSSYDYETESSTYAMAAPSSSATCSSPMAAAAGDGSLFNFPGGVSS
DHRYWMQFLSSDDQAFRFHNNPSHRFMLPPVEFHQSSFVPEYANHDEILSVAHHH
HQHQAASGFSDDYFPFVPQNNEELADMDSSLLTEQLNQDDQQNVGEELGSDRMV
ENLRWVGVLDKDIEKVILDHSAILV*
>itf03g06200.t1.
MEVQLPGFRFHPTEEELLNFYLRSAVNAKKPRSDIIGFLNIYQHDPWDLPGKASEG
QGEREWYFFVARDRKHGGGNGKANRTTGNGFWKATGSDRPIRSNSPSEGRKVLG
MKKTLVFYQGRAPRGRRTDWVMNEFRLSDNTTCSLKEDVVLCKVYRKATSFKEL
EQKSLKEEQGYAVAATPTPASGGYGVHLKDFESTFFHVPTISSDQESNHDKAQKFS
ALYAKHGFVQQEDALSEEVDSLGNLFQVRDNWSLYAMY*
>itf03g07030.t1.

MDTADESCTVPPGFRFHPTDEELVGYYLRKKVASQKIDLDVIRDIDLYRIEPWDLQ
DKCRIGYEEQNEWYFFSHKDKKYPTGTRTNRATMAGFWKATGRDKAVYDKAKLI
GMRKTLVFYKGRAPNGQKTDWIMHEYRLESEENGPPQEEGWVVCRAFKKRITGQ
AKSMEGWESNFLYDEPNINNMDYMTRQAAAAAAPSNFMAAQTFLCKQELEASEN
LTFMHADQLFNQLPQLQSPSLPLLKTPLLHENHRSNATEDTASAAGAGEQIKNNIN
CVSNKKVTDWRALDKFVASQLSHDVNQESFEDHGCDEMGSLLLLQSEMRDNGG
GGGSGGRLNDFLNSGSSECDNIGICIFDK*
>itf03g18330.t1.
MNKNSSQVGSISSSDLIDAKLEEHQLCGSKQCPGCGHKLEAAKPDWVGLPAGVKF
DPTDQELIEHLEAKVVAKESKSHPLIDEFIPTIEGEDGICYTHPEKLPGVTRDGLSRH
FFHRPSKAYTTGTRKRRKIQTECDLQGGETRWHKTGKTRPVMLNGKQKGCKKILV
LYTNFGKNRKPEKTNWVMHQYHLGQHEEEKEGELVVSKIFYQTQPRQCNWSSAA
AATAAERSITSGGEDPASKRDSGGSGSCSSSKDNNNMNIHSHGDELSVAVGAAMS
SYSAMEIQQLKATADHFSFLPFRKNFDEAGIAVGAEASMGREVPATGTCEGRDIAA
DPHRGHHHVTHDPHHPHHHHHQIAAAFHNISRPSHTISAIISPPPPLHHHASVILDED
PFHVPRIILPADNYQQSQQQQQQQHHKLGGRSTSELEELIMGCTTTSSDIKEGSSM
AANQQEAEWLKYTPFWPDPDNPDHHG*
>itf03g28670.t1.
MKGNGGIHDSKQSQLELPPGFRFHPTDDELVVHYLCRKCAAQSISVPIIADIDLYKF
DPWQLPEMALYGEKEWYFFSPRDRKYPNGSRPNRAAGTGYWKATGADKPVGKP
KTLGIKKALVFYAGKAPRGIKTNWIMHEYRLANVDRSAGKRNNLRLDEWVLCRI
YNKKGSVEKYYVDEKEVNFPELEDEKPKITTPNILQNEVGLPPVIPVNNQNGYLQL
DRSESVPRRQTDSSCCSEQVQSPGDKEVQSAPKWDDLNFQFNNFSDIALQTTFPFN
EQHFNPFQQDILLMHMQHHKSPFNFC*
>itf04g18350.t1
MCFHPNVDQYGNICLDILQDKWSSAYDCRTILLSIQSLLGEPNTENPLNSSAAALW
KNQEERSTTVLLPLSSLPLGFRFHPTNEELINHYLKLKINGLKAEVSVIRKIDICKLEL
WDLPGESYAWLKMLWRKLEMQVKAMLTLRNQCVKPLTLLGEERRLELKLDPMA
SCHIVKSSKNVIIIKRKWAIVRSKGSTEEEKETLT*
>itf04g21110.t1.
MEMECCVPPGFRFHPTEEELVGYYLKRKINSLKIDLDVITDIDLYRMEPWDIQDRC
KLGYEEQSEWYFFSHKDRKYPTGTRTNRATAAGFWKATGRDKAVMSKEEQIIGM
RKTLHPPPHTHTLEPGHCHRWATLKSNIRRAAQLTRNNKSNSN*
>itf04g26240.t1.
MGLRDIGATLPPGFRFYPSDEELVCHYLYKKIANEEVLKGTLVEIDLHTCEPWQLP
EVAKLNSSEWYFFSFRDRKYATGFRTNRATTSGYWKATGKDRTVMDPRSQAIVG
MRKTLVFYRNRAPNGVKTGWIMHEFRLENPHIPPKEDWVLCRVFHKSKGETSENN
NLNTCMYDNSIVLPAATSPPSLAASPPNMDAASFPYRPIAAAACQNQSATTAAAAN
FFNVSLSVPEHHQNGFLRPSHEMPHSNKCETDQYGFLFDMNFEEPNFHDGGVHSSL
DDMRFDDENGMVFI*
>itf04g32060.t1.
MGAEIISPGFRFYPTEEELVSFYLHTKLEGNRPDIDRVIPALNIYDLEPWQLPRVSGE
LCAGDMEQWFFFVPRQEREARGGRPCRTTATGYWKATGSPSYVYSSGNKVIGVK

KSMVFYNGKAPCGRKTKWKMNEYRAIEEVLLPATSSSTATVPKVPNNQLKLRWN
TYFNLYIHNYQLRLLVEMEHMLPLIYILKVKC*
>itf04g33330.t1.
MEEFLVPDPTFVHDENTIVVRFCPSDEELIHSYLWKKVQNQPLPPNKIHEVNLYKFS
PWELSEMFETIGEKYWYFFTNRKYKNGQRSNRAAGSGFWKATGADKSIMNKQKV
LVGSRKALVFYEGKPPKGVKTSWIMYEYRVEGALSPRPRGDDDWVLCRIYNKTD
KSPEKKAAPKLSFLSKQDEMDEMPAFYYLPMNEMPASPDTKLKQLDCTYYIYAHN
VITNEPFQVADLENLFITENDDDSIQKPME*
>itf04g33340.t1.
MEEFLVPYHTSSAPTIFVGPSSDIAIVHDENTIVVHKSNNRLINNNSFDDTNNDDGF
NFPPGFRFCPSDDELIECYLWKMVQNQPLPPNKIHEVNLYKFSPWELSDMYETVGE
KDWYFFTPRDRKYKNGQRPNRAAGSGFWKATGADKSIMNKKKVLVGYRKALVF
YEGKPPRGRKTNWIMHEYRVEGAPSPRPRGDANDMRLDDWVLCRIYNKTGKSSE
KKLGKKFSPPPVEQPAAAQAVAVVSPSLLPPPPQIEPGMPPLIGDEFALAQFSFLSG
QDEMDELPAFYYLTMENDMMIPNDQPFHTANLDNLFITENDDDSIPKPME*
>itf04g33740.t1.
MVVQPFHFPPFNFPPGYRFLPTDEELIVHYLKKKIMNYVLPHNQIQEVNLYKYSPEE
LSAGRYPKLGEKDFYFFTHRDRKYKNGNRPSRAAGDGYWKATTADKPITHGVDG
PVIGHKKTLVYYEGRPPKGEKTNWIMHEFTVEEGTITPKPRGDNVDPMRLDDWVL
CRIHKKAAGKVGKSTQKEDENINIVEANTSPPQDEQSEEVQGMQFSLPLNEVVQG
VQFSLPLNEMFTFHDPFVFGNGPAYNDPQFINSGLFIPENIYNQTFDFDIDHFLSSQN
GFPEVLHFYAIHPTVQKPFAFSPIQMVLSNIHRLAHRLSLHSVAGKLSLLSVCRGYS
TEKCCIPNLLIDSNSSNEYRSARECYRLLGLSRNATRLLTKGPSHWRHEHFCTAAG
NNDPGNASQKEKISVTFVDKDGEEQQIKVPLGMSMLEAAHENDIELEGACEGSLA
CSTCHVIVMDMEYYNKLEDPEDEENDMLDLAFGLTETSRLGCQIIAKPELNGIRLAI
PAATRNFAVDGFKPKPH*
>itf05g00220.t1.
MSGFCGSNDYVQSSPVEKDSGDSIRIPPGYRFYPTDEELINHFLRRKIEDPSFFTTAIE
EADLKRLEPGVCVGVRKRFFFSRRDMIMKYGTGQRATDSGYWKTTGEDREIFKG
KTLVGMKKTLIFYRGRAPSCERTNWVMHEYRLEGHNSLHNLPQNAKNEWVICKV
FLKNPLEKKTFAWDIHTLFVMAIQSLAWAYEVGKHYVNIKRKDYNNEYVGGVRQ
LQPKLHKSNSDAENPAAAAASSSMNSHNHNSNNTTMEDNSRVCVKVVGYENDTV
MFLLPFPTMDSLKAEILKRFNNLEAETFKIRYKDEDEEMVTIACDEDLHYCLEFFKS
TGTTPVRLSLLI*
>itf05g00240.t1.
MSCFCGSNDSVESPPVEKDFGDSIRIPPGYRFNPTEEELITHYLWRKIANPSFFTTAV
EEADLNRLEPWDFPGVCVGEGEWFFFSRRDMKYATATGQRIHRATDSGYWKITG
NDRKIFKGKTLVGMKKTLVFYRGRAPSGERTDWVMHEYRLEGHNCLHNLPQNA
KNEWVICKVFVKSSLEKKAYISGIQELKLAGMHTLFLSLIFLAVQNLAWASNDSST
NEGEVGKHNVNCCRSSTHKRKDNNNEYVGVRQLQPKLQKSNSDAENHPAAAASS
SMSSHNNITMEDNSSVCVRVGYNNDTVKFLLPFATMDSLKAEILKRFNKLEAKTF
KIRYKDEDEEMVTIGCDDDLHYCLEFFKSTGTTPVRLSLLKESIAPSLEHV*
>itf05g00910.t1.

MEKMSFVKNGVLRLPPGFRFHPTDEELVVQYLKRKVFSCPLPASIIPDFDVCKSDP
WDLPGDWEQERYFFSTREVKYPNGNRSSRATGSGYWKATGIDKQIASCRGRQLV
GMKKTLVFYKGKAPHGSRTDWIMHEYRLANAPTSQHPPNNNNENWVLCRIFLKK
RPGKKEDEETEIRGAASLGNGTKPVFYDFLARERADLNLAPASSSSGSSGVTVLSS
NHQTEDHEESSSCSSFTTLRTKP*
>itf05g01820.t1.
MEMMECCVPPGFRFHPTEEELVGYYLNRKVNSLKIDLDVITDVDLYRIEPWDIQER
CKLGYEEQNEWYFFSHKDRKYPTGTRTNRATAAGFWKATGRDKAVLSKEKIIGM
RKTLVFYRGRAPNGKKTHWIMHEYRLQSSEHAPPQEEGWVVCRAFKKPIPNSKPA
GYEAWNNNNHCSYYVRDNNNNNNSSYTTTLNASNNTPNHHHSPVINPTFNHQIGT
NFPQFPFTPPHLNPGGGGYDQINHQQLVELPQLDSPTVSTKDGAAAAATASEDYED
VVDRNSYGGGDHSWKDLDYKMIAPPQGVMMNDHPVLPSYSFANMPLLIRDDDR
NHFSHLLECFPDL*
>itf05g09150.t1.
MRCESNFPEIILFDEEKCAVAAAAGNRRKEAMNKSGSDLIDAKLEEHQLCGSKHCP
ECGHKLEGKPDWVGLPAGVKFDPTDQELIEHLEAKVLARDQSKSHPLIDEFIPTIEG
EDGICYTHPEKLPGVTRDGLSRHFFHRPSKAYTTGTRKRRKIQTECDLQGGETRWH
KTGKTRPVMVNGKQKGCKKILVLYTNFGKNRKPEKTNWVMHQYHLGQHEEEKE
GELVVSKIFYQTQPRQCNWSAERSSTGAAASEPTSCSSSRRDELSAAVVGAPMSSY
SAMDIQQLKAEHFGFLPFRKNFDEAGIVGGEGSRAMEGAAAAVPEQGGACEERDI
SDQHQSHAYHVTHDPPQHQHQHHHHHHQIAATQFHIISPPPPLHHASVNILDHNID
PFQVPRMLLPNDNFQCFWQQAQQQQQEEHHKLGGGRSTSGLEELIMGCTSSDM
KEETSIPNPQEAEWLKYSSFWPDPDNPDHHG*
>itf05g12720.t1.
MVMAKEGLPDQRLPPGFRFHPTDEELVLYYLKRKICRRRILTDVIGETDVYKWDP
DDLPELSKLKTGDRQWFFFSPRDRKYPNSGRTNRATKHGYWKATGKDRTITCNSR
DVGVKKTLVFYKGRAPTGERTDWVMHEYTMDEMELSRCQSAQDYYALYKVFKK
SGPGPKNGEQYGAPFNEEDWADDECVGTNLHVEQEKSGKNVNDVGLIDDPKPND
VLPPPIDGLEEFLKHIADEPSLTELLPVDNSYVDKLVGNEDTASVLLDHAAGEVYLP
VQTATLFSVPPLHNEQTIFDPIHSGTLQPQLYEATEVTSAPVNERNPKVVEEDFLED
FLEMNDLDGPELSVNQNFDKPVENLGSQQFVDFDGLNEFELYQDAAMFLHDMGA
IGVGQGSEQAANHVANGIINPLSTDFGDQMINNQQSYLNEGNEFNHQLWSHDLRC
STVNPTEASQNANLPLTSGVVYGNHLENHPVGANLNQNNIQDDATNSSFSSALWA
FMESIPATPASAAESVLVNKAFERMSSFRRLRLNARNVKVASGNTSATSRRSGKSK
NGLYRLICFSLLGVLCAILWIFIGAS*
>itf05g16070.t1.
MENTSGSAFSGVEEEMELQMPPGFRFHPTDEELISHYLCPKVADSSFCAAAMGEV
DLNKVEPWDLPWRAKMGEKEWYFFCVRDRKYPTGQRTNRATEAGYWKATGKD
KEIFRAKTLVGMKKTLVFYKGRAPRGQKTNWVMHEYRLEGNYSIPNHKNEWVIC
RVFKKTAGGHKIHISGLTRGENLPALMEVSQRTATSGEAMAPHVTCFSDSKEDKK
PIIAPPHFSSPSPSSKNSDFSGVFPGLEFQHYSEPDENSVLRLWLENNGLQAKQGND
DDEFLMSSANGPVDLDCLWNY*
>itf05g20060.t1.

MAVLKVERPTGLPPLHSLPVGYRFRPTDEELVDHYLKMKITGSEAEVSVIREVDIC
KHEPWDLPDMSFVESHDNEWFFFCPKDRKYQNGQRLNRATERGYWKATGKDRTI
SSRKGAKVGMKKTLVYYLGRAPEGKRTYWVIHEYRTTEKAFDGTHSGQAPFVLC
RLFKKVELKLDEGAEISNSGEVDPIVASPTVVKTPTDDEQSEGGTPLMKSDHIKTQP
LTPGQSSVEEAPGVHFPIDSNSNSCIADTTEDQVLDITSNPADPDLEPWKFYFDPSA
GPIFSPTYEQMPEFGSSYFYGDVINCISNNNDVQFQYGTNGFDPNEFLNPAALVSSD
GESEEVTQMLAEPVGFENELFATNNQEALLQRQVNYVANSLVGTFATPTMGSDH
QNWNLDLQNNNYLGQSLFSSVSTGTVAPQMVFPQAETVGNISSSGTGITLRTRQPQ
NQRGDQQSNAQGTAPRRIRFSTKFQVGPVQCTRPQTTPEDNTVSKEDASTVDKPNS
TASATAQDPILEGRGEDNTGITTSKKEGDTSACRKETSVKASSKTALCSASSSIYIPK
VLVVVSLVVVFVGAWACFVRL*
>itf05g21000.t1.
MNSGGIQFNRRPELDFLPPGFRFHPTDDELVLHYLCRKCASQPISVPVIAEIDLYKFA
PWQLPEMALYGEKEWYFFSPRDRKYPNGSRPNRAAGLGYWKATGADKPVGKAQ
GIKKALVFYAGKAPTGIKTNWIMHEYRLANVDRSAGKGHTLRLDDWVLCRIYNK
KGILEKYDNVVAADDQKPKITGFSP
LAPPQSNQSEYLHWHTDSSSSSDQMLSSPELAPEKEVQSSSGWDDLDFQLNNFMA
AFPADDPFNEHFNSFPDILFPLQ*
>itf05g23010.t1.
MAPVSLPPGFRFHPTDEELVAYYLKRKINGRKIELEIIPEVDLYKCEPWDLPGKSLL
PSKDLEWYFFSPRDRKYPNGSRTNRATKAGYWKATGKDRKVNSQMRSVGMKKT
LVYYRGRAPHGARTDWVMHEYRLDERECEIQNGLQDAYALCRVFKKSLTNTGPK
IGDHYVSAASDRSSSMDIYSEGRCEEMESSNYAMAAPPSSTAASCSSMAAAAVHG
SPFHHVAAAGPSTNDDKWMQYLSDEAFSFHTPPPSSLPNYATMPYPPSKVDIALEC
ARLQHRFMLPQLEVQDFPHVVGHVDARMIPHHHQSSFVNHDNNPDIVQEILSVAQ
ASQDLMNQDGWGGGGYAPTAASDDDFSFLLPHNANQFQDMGNFRFMDQLREDQ
NGGRNIEITDFGDEFKPDRMVENLRWVGMSDKDLEKTLLEDYKAVPIENVSGFNR
EGHEDHGESSQHNNFSDINDFSLGFDNDHDHNHNANNDNNNFLEDFSGSPSFEVY
EKVEVKHGMFIASRQAAKTLYHQVVPSTTVRIHRNLLVPMQAATFPISKFEAPRAF
NNNNNNNNIVLDKFIAFSRKMLIGIIKLTRPWGRMVSTLVGVIAILHTCWVYFGELLL
EAQEKNYYYCHFSGEKMKRELSGGDYEWENLKKNKDSSAIADKKNPCRMTTRV
DKTTWPYLTLVLALSTIWLHQNVTFF*
>itf05g25290.t1.
MGVKETDPLSQLSLPPGFRFYPTDEELLVQYLCKKVAGHDFSLRIIGEIDLYKFDPW
VLPSKATFGEKEWYFFSPRDRKYPNGSRPNRVAGSGYWKATGTDKVITTEGRKVG
IKKALVFYVGKAPKGSKTNWIMHEYRLSDAPKKTGSSRLDEWVLCRIYKKHSSAE
KAISGGVPTKEHSHGSPSSTSSQFDDMLVALPELNDQSFVLPAMNNLKQEEQVDLH
ELGPMSFDWNTISAAQPTLPDFSAGAGAGNQVSAAQVTGNEVNYQNDVYAHALA
MNFQVPDDEVQSGLRSFFLPQSFSNSPDPFGIRYPTQSPSMGFRP*
>itf05g27190.t1.
MSSDRTKEATKLIHLSIMEEAENLASDKHNQFQEHDDDDMFLPGFRFHPTDEELVG
FYLRRKVQNRLATLDFIKQTDIYKFDPWDLPKTASSVGENEWYFFCKRGRKYKNSI
RPNRVTGSGFWKATGIDREIYPAGAGQRESRHCIGLKKSLVYYRGSAGKGTKTEW
MMHEFRLPPQHHKSSAAAHPKNIAQEAEVWTLCRILKRKSMLEWKELTAAKRNS

SNPSAETCMRSKESLSSNMENYISFSNPVNCNVNNVTKHSHENEERPFGISVNVGR
TDNVDYHIIYGSHQQQQQQINNQVSQLSHYYPPYPPAANSLSSFTSATPEIMNGFPK
HADWEELRSVILGFATHP*
>itf06g13280.t1.
MAIVPSRMSQQDESSNNNNNNHRRHEEDDEETDDHEHDVVMPGFRFHPTEEELVE
FYLRRKVEGKPFNVELITFLDLYRYDPWELPALAAIGEKEWFFYVPRDRKYRNGD
RPNRVTTSGYWKATGADRMIRSENFRSIGLKKTLVFYTGKAPKGIRTSWIMNEYRL
PHHDTERLQKAEISLCRVYKRAGVEDHPSLPRSLPTARATPSTCRPPPHAAKKPPH
HSLPPFEFPRTTANMEAAATSLVLSQPTNSMNSYTPAPPAPPLAAVVASEEEKLLLT
SPVVFPGGSSSPAAPFAAVDDLHRLVNLHQAAAPQHFLTPQFSPLQLQPPLTFPDRL
WDWSSLAPPPEQNKDYGASNPFK*
>itf06g14060.t1.
MEGNLPPGFRFHPSDEELITYYLSNKVSDFTFTAKAIADVDLNKCEPWDLPGKASM
GEKQWYFFSLKDRKYPTGLRTNRATEAGYWKTTGKDKEIFCGGVLVGMKKTLVF
YRGRAPKGLKTNWVMHEYRLENKHGFKPAKEEWIVCRVFEKSSTAKKPQATSSSP
QSPESPCDTTTFASELEDISDPPNLSWQNNNYFNTPITTLPLLPWPPALLTPTTNLSS
VNSMLFRALQLRAANQARDQQAPIATTFGYSHTPQGTENHHHHLLTQFENDFNNF
AVTTASSSMVLDSVNQQEPPLEQPYRLDSNIW*
>itf06g14780.t1.
MAGGLQLPPGFRFHPTDEELVMHYLCRKCASLPISVPIIAEIDLYKFDPWDLPDMA
LYGEKEWYFFSPRDRKYPNGSRPNRAAGRGYWKATGADKPIGQPKPVGIKKALVF
YSGKAPKGEKTNWIMHEYRLADVDRSARKKSNSLRLDDWVLCRIYNKKGELEKA
DVSIRNMSKPTASSPVSEEEEKKPEMMLPPPPQPQPPQVFSDFLYLEPSDSIPKLHTE
SSCSEHVLSPEFTCDREVQSEAKMSEWDKTALNFPFNYQNATADGGFLGCGDLPG
SYDAWPLQDMLLYKPF*
>itf06g15150.t1.
MADDAENLSRSVITKVDEEEDDDVPVVLPGFRFHPTDEELVGFYLRRKIEKRELSM
ELIKQIDIYKYDPWDLPKSSHVGDTEWYFFCKRGRKYRNSIRPNRVTGSGFWKAT
GIDRPIYSSSAPAGENHRHCIGLKKSLVYYRGSAGKGTKTDWMMHEFRLPADHDK
STKHIDIKSIAQEAEVWTLCRILKRSVSHKRDWKEISAKKSNNHYTNVNSTTYNNM
ESCCDDQKNNYISFNNAPLMANHTTNNVCHHLLTAQLNPISQPQFLSYSTTANAY
YSPVVAAPDIDELLKHEDWDEITQLF*
>itf06g15330.t1.
MNSGGSLSMVEAKLPPGFRFHPRDEELICDYLAKKINGSGDPHHLRPPFLIEVDLNK
CEPWDIPGIACVGGKDWYFYSQRDRKYATGLRTNRATVSGYWKATGKDRPVISK
GTLVGMRKTLVFYQGRAPKGRKTDWVMHEFRLEGPLGPPPLSSTKEDWVLCRVF
HKSKEIPGAKQGIVSGGGYNTTYDHHHDGVAITSSSLPPLMDPYGGISFNQTHPNN
ATALLNDYYEQVPCFSIFSPNNPAATAAATSAFHNPAAAATTTAFNALPPDICAYF
NSASSSSTDKNVIKAVLGHLNINYNNNNNNNNNTIINNNNVNNMVNVKGGGSPSF
GEGSSESTSFLSEVGLNSIWNNDY*
>itf06g19310.t1.
MAAADEQTVAAAADEQPVVAAMEEGMAAEEQQVVAAIGEGAQEHAQAQAETV
TPFVAVMGEGSQAQAQTEAPFVAAMGEGSQAQAQAEAPFVAAMGEGSQAQATF
VAAMMPKPKLAYGFKFHPTDEELVVYFLRRKVLRRPFPFEAIAEVDIYNFEPWELQ

GFCAVDSREAEWHFFTRTEKKYINGSKINRATINGFWKATGRDRAVHHKSQIVGL
KKTLVYHAGRAPAGKRTDWLMHEYRLSEQELDKTGVQQKDAYYICKVYLKKDV
MGRRGVPFNVEDWENHTGILIPCEDNNAEDEASEVNLQGASTSKAVQLPEKAQGF
KSLRIRAREEDDDDTDDDDDETEDDEAIVCLDKGKKPKLMLDFFSGAGGKGDSDD
DSSSATISDSLTPDFSSGPLNFSLTAKPKEKENHGSTAPNNAGGAFNSSCLENSLPPG
YLKFITDLEEERDSLKHQLMNSEATVSCLQAENEEMAEEIMRLRSLLPGQGSD*
>itf06g19320.t1.
MEKQKAAALTGGNLDLALAPPTRRLGPGFRFHPTDEELVVYYLRRKVRGKPFHVE
AIAVVDIYKHEPWELHAFSAVNSTDQEWYFLVDLEKKYKGSRLINRSFTEKGYWK
TSEKDQTVSHKREIVGMKKTLVYHAGKPPNGRRTNWVMHEYRLVEAELDKARV
QQDDSFILCKVFCKNEMGPPCSGVYGPFNEEEWENDEVMRQAGFTSNTKNRLKFD
LFPGNKCVSSDEASSLSSDVPQPGLLNFSLSAQPNLGNHSHLPPKVVGAFDSSCLEK
SVPPGYLKFINDLEEERNKLVDSMKNSEATIRRLLESNEQLEEENRRLKNLHF*
>itf06g26120.t1.
MENLPGDDNQMDLPPGFRFHPTDEELITHYLSKKALDTSFCAVAIGEVDMNKVEP
WELPWKSRIGEKEWYFFCVRDKKYPTGMRTNRATASGYWKATGKDKEIFRGKCL
VGMKKTLVFYQGRAPKGEKTNWVCHEFRLEGKLSVHNFPNAAQSDWVICRVFHK
NSCGKNVHFPDTKSSVLPPLTDSSTTTTPPESSHVHCFSSLIPPEHKSLQQESMNMV
NYLSNINPPLPSSSTADISPHPQTVFPWSQAVQTHGSTFLQNPDSFPIQDPAILTSLLA
GCGHRSFKTEKEMVCGSQETEISSVVSNLEMGRRAFDDHQNPQELDCLWSY*
>itf07g00540.t1.
MAEITPGFRFYPTEEELVSFYLHNKLEGTRQDINCVIPMINIFDLEPWHLPKVSGELC
VGETEQWFFFVPRQEREARGGRPCRTTAAGYWKATGSPSYVYSSDNKVIGMKKS
MVFYKGKSPCGRKTKWKMNEYRAIEEQLPSHLHPSYSSSLPFQKLRHEMTLCRVY
VILRNFGSFDRRPIGNMASSKYGTSQEHGVNSQEVKMIPMEAIKCSESSVLQGEEEE
EDFDDGMNVYATTGLTRMVKSPEPFSGLDRLINFQLSNWNE*
>itf07g00590.t1.
MGDGHASSSRNSNNNNSTEIPLATASAMFPGFRFSPTDEELISYYLKKKLEGSDRC
VEVISEVEIWKHEPWDLPAVKSVIQSDNEWFFFSPRGRKYPNGSQSRRATESGYWK
ATGKERNVKSGSNVIGTKRTLVFHTGRAPKGQRTQWIMHEYCMNGRSQPQDSMV
VCRLRKNNEFHLNEAPRNQMNNSNANNSMTALSGADPAGSSGGVNAGESCSKEC
SSSFNSHSVEQIDSDSDSDSDEKLVNEISLHHPPGHQKDFFDAEDWFADIMKDDIIK
LDDSSLNPRLDALPATTTKPEPEPQQNQATAAQGFTPDLFPFQGTANRRLRLRKEA
ARGEPFQLDKQQLLPRRLVSMMSRGRIKHCSIYVCFLSLIILLFCCSGLLLRLENLM
NK*
>itf07g03260.t1.
MASSSSGGVPPGFRFHPTDEELLHYYLKRKVSYQKFELDVIREIDLNKIEPWDLQER
CKIGSTPQNDWYFFSHKDRKYPTGSRTNRATNAGFWKATGRDKCIRNSFQKIGMR
KTLVFYRGRAPHGQKTDWIMHEYRLDDADDPQANPNEDGWVVCRVFKKKNLFK
VGSESGGSAISDQLNTTAANQSRAAAAAGFSLRDTAPYLLQPPHGHGGFNYSHHIP
LPHHYHQLEPQTFIPAAAHFSDHHAAAAGAPIMVKQLMSDPTRDCESGSDQNLCY
QPATAACDSGLEVGSCEPPPPPQSMSEWGMLVSSQMGQDDHGSSKDTRVGFEDA
NGSSMNHINPLSLRSEMDFWGYGK*
>itf07g11480.t1.

MASRPAISSFHFPPGVRFHPSDQELIVYYLFNKVNSHPLPACIITDVKFYNYNPWDL
PKLAIFGEDEWFFFSPRDRKYPNGVRPNRTTASGYWKATGSDRPILSPDGSKIGVK
KALVFYIGKAPSGLKTDWNMMEYRLPDTSIKPSRPKGSSMRLDDWVLCRVRQKG
NMSKNAWEAQPSPQELLSYLPNNEKLPSVNDDPLRDINTNYLLTKDNSLIYSMFLQ
GLIPLENILRAASQFSSDGKYYTGYEHESGKVNTQVTNSSFQSFFGSSKRKHDDGT
KYQNPHDLIKTISDGNKSEDFLLSNMLATNHRNFYNENQPPVDTFSGIQYQMP*
>itf07g17390.t1.
MGLRDIGATLPPGFRFYPSDEELICHYLYNKIANQNVSKGTLVEVDLHTCEPWQLP
EVAKLNSSEWYFFSFRDRKYATGFRTNRATTSGYWKATGKDRTVIDPTTRAIVGM
RKTLVFYKNRAPNGIKTGWIMHEFRLENPHIPPKEDWVLCRVFHKSKTETNNEHNI
MYDDMASGNLEVCLPSTAAYRPVAAAYSHSHSPPNQTQTSSTANFFTGAPADQRG
FPQSSRHNTNVAPSSNDYEFLFDDMNFEDHPSMQDGGVHSSIEDMRFDDENGLAFI
*
>itf07g17700.t1.
MVPLERSTTKVLLPPSSLPVGFRFHPTDEELINHYLKLKINGSKAEVSMIREIDICKL
EPWDLPDLSVIKSYDNEWFFFCPIDRKYQNGQRMNRATELGFWKATGKDRNILSK
KRVKIGMKKTLVYYEGRGPNGKRSNWVIHEYRATGETPFVLCRLFKKNENGESPN
NNGVEPNVSSPIVVKSPGEDDQSDRFNANNAEKYVDYIVHDHDLEEALAYLYEPL
EEDFGIFSLPQTEMEGLGFSYAVTNDFGDDMDIQSLSETNISDENEFMNSFLVSSDEI
PYANGSI*
>itf07g21970.t1
MVAKNRSSSDLPPGFRFHPTDEELIMYYLRNQATSKPCPVSIIPEVDVYKFDPWELP
DRAEFGENEWYFFTPRDRKYPNGIRPNRAAVSGYWKATGTDKAIYSGSKYVGVK
KALVFYQGKPPKGTKTDWIMHEYRLSDSRSHPPKQNGSMRLDDWVLCRIYKKKS
MGSNRAMEIVKVEEETQLPQVNIATNCEPLIGSGPQGLILPRPCSLTHLLELGYLGSI
PQLLGEDDNNNSNFHNYPNLDHHQSPFMGSSGNNVPFALGESTPQHQYFSSQSGFS
SNQPMPIFVNPVAFQFQ*
>itf07g22170.t1.
MDGRSSDQTEKIMDEVMLPGFRFHPTDEELVGFYLKKKVQHKPLSIELIKQLDIYK
YDPWDLPKLAAVGEKEWYFYCPRDRKYRNSARPNRVTGAGFWKATGTDRPIYSS
ETSKCIGLKKSLVFYKGRAARGIKTDWMMHEFRLPSISDISTPKRCLDKNIPPNDA
WAICRIFKKANSNAQRALSHSWVVSSSPPTIPQTPTTTTTQQTLLTQNPNNLNQVS
MATHDFKNSPEIMGLDLLPYNDPSTSYTFPPSLFTHPPLQKPTINDASSLLLDMSNS
VFGEFVGKGSGESTVDYGGLINDGFSVVGPENAVEGSLQDGGGGGGSGGAVVVV
GKESSNIIDESIRFSFNGFPLNMGAENAWKLSSLVWESSPCPSEISSTSYSTSKCYT*
>itf08g01730.t1.
MGGASLPPGFRFHPTDDELVGYYLKRKTEGLEIELEVIPEVDLYKFDPWELPDKSF
LPKRDMEWFFFCPRDKKYPNGSRTNRATKSGYWKATGKDRRIVCQPATVGYRKT
LVFYRGRAPLGDRTDWVMHEYRISDEDSHGNPSFQGPFALCRVIKKNDISKKTTSE
ASGVTAAAAASKETGSSSSHAGFSSSVALNEPIILSDDIPTQTAFMSTESNYSTPIASP
YQASHMGDYEFGMQPDTASVWMSADMILDSSKECPQQQNVPGFHSTQYGFPGST
LWQPYENHELSSSSSYSNFRGEVELSDDPSRYGCVSPYFGHGNYLGFYGNDGRPFE
GYDQNSSSRNPNLF*
>itf08g04440.t1.

MGDVNNDTTSFALPPGSGFYPSDEQLVRYYLSPKNDGDRHGAANVIKEINLYNFD
PFNLPETSCFRFGRGGRRRHWFCFVARALKDRGIRRAGGGYWKRRGRDRVVVDR
RLGKAVVGKRKCFVFYFGDSQSPKTAVKTDWFMYEYELLNHPTAAFVLCRIFSKS
HHGNDLSENMVSSCAEESFASVRNIGIQCNGTATSATEEKMQDEKNDALNFPVDT
VSKIDNLVSEPIGDQMRSTGLVSTGATFIEALAAEEGLGILEGDFLELDDLLCPLLG
VD*
>itf08g07600.t1.
MNLSVNGESQVPPGFRFHPTEEELLHYYLRKKVASQKIDLDVIPEVDLNKLEPWDI
QEKCKIGSTPQNEWYFFSHKDKKYPTGTRTNRATAAGFWKATGRDKVIQSNCRRI
GLRKTLVFYKGRAPRGQKSDWIMHEYRLDDIITSHDATGTNLVGEMSGPDEGWVI
CKVFKKKLYHKAAMEYNHHPQTHQRNDGVLDQILTYMGKSSSSKHPHAGETKNI
LLAAAGENSPPQFLHMPPLETAADISHIIPFDDMLADTEPFYPCYDGGAGAGVDFS
VDCYGAHPTVDDLRPYDQNQISQVHSTLIMDPLSHLSV*
>itf08g10040.t1.
MDNLSLLFPGFRFHPTDEELVLHYLNPKVLNSPLPAAVILEIDNVCKSDPWDLPGG
SDGERYFFSMREVRNRCSRATLSGYWKATGKDKKIVVSGEAQSVVGIKKTLVFYK
GKPSNSSKTDWMMHEYRLGDVPQPQANWVLCRIFLKKRGGERSRAASLIPTFRDF
FAPERADLNLAPASSSSSRSSSVIESCNSQTSDHQEATSC*
>itf08g10050.t1.
MDNLSLVFPGFRFRPTDEELIVHYLNPKVLNSPLPADVILEIDNVCKSDPWDLPGGS
DEERYFFSMREVKNGGRNRCSRATLSGYWKATGKDKKIVVSGEAQRVVGIKKTL
VFYKGKPSNGSKTEWMMHEYSLGDVPQPQANWVLCRIFLKKRGGERSRAASLIPT
FRDFFAPERADLNLAPASSSSSRSSSVIESCNYQTSDHQEATSC*
>itf08g10060.t1.
MDNLSLVFPGFRFRPTDEELVVHYLNPKVLNSPLPADVILEIDNVCKSDPWDLPGG
SDGDRYFFSKKEVKNGGRNRCNRATLSGYWKATGKDKKIGVSGEAQGVVGIKKS
LVFYKGKPSNGSKTDWMMHEYSLGDAPQPQANWVLCRIFLKKKGGERSRAASLV
PAFRDFFATERADLNLAPASSSSSRSSSVTESCNYQTGDHQEATSCSRAVL*
>itf08g10090.t1.
MDNLSLLFPGIRFQPTDEELVLHYLHPKVFNSPLPAAIILEIDNVCKSDPWDLPGDL
DGERYFFTTREVKNGSGNRFSRATLSGYWKATGKDKKIVVSGEAQSVVGMKKTL
VFYTGKAPNGSKTDWIMHEYRLADAPQPEENWVLCRMKKRGGRNGERSHSSSRD
AFHDFFAPERADLNLAPTSSSSSRSSDHQDS*
>itf09g00190.t1.
MEESRLEMELPGFRFHPTEEELLEFYLKNMVLGKKLRHNVIGCLNIYRYDPWDLP
GHANNVGEREWYFFVARDTRHGSGGRPNRTTEKGFWKATGSDRKIFSLSHPPNR
MIGLKKTLVFYQGRAPRGCKTDWVMNEYRLPDALLPPSSAKNIVLCKIYRKATSIK
VLEQRAAMEDEINTHASPSFMTIDTTLSCSDHHHHDLVAQSFLTVTMNNNNNNKE
EDDDGEYSTGTMLTVDGEKAAAAAAEGEFSKDKLQFLPTSNSNSNSNSNSITTSGS
SLLLSLQSGEEVKPLLLPTKLELPSAAAKSSSDYWASHLQDPFWAQLRSPCLLNDLI
ALTPLPPNLHNF*
>itf09g03660.t1.
MGENSVNLPPGFRFYPTDEELVVHFLHRKAALLPCHPDVIPDLDLYPYDPWDLEG
RAMGEGNKWYFYSRRTASRITSNGYWKALGGDEPIFPSSGGHRVGVKRCYAFYIG

EPPQGDKTSWVMQEYRLSASPDSASASTTSAKSSRRRTHSKNDYSKWVICRVYDS
NCDDDGDDGNELSCLDEVFLSLDDLDEISLPH*
>itf09g06980.t1.
MTWCNNSAAGDQPPLQIIPSDDDNAAAATSRIRSVTCPSCGHSIQLESQGIHDLPGL
PAGVKFDPSDQEILEHLQAKSVSDSRKLHPLIDEFIPTIEGENGICYTHPEKLPGVNK
DGQVRHFFHRPSKAYTTGTRKRRKVHTDADGGETRWHKTGKTRAVFGEANLRG
YKKILVLYTNYGRQRKPEKTNWVMHQYHLGHDEEEKDGELVVSKVFYQTQPRQ
CGSSSSSSSKQPLNKTSSVSFSRRDDNNFVDYYNPPFISSYHVGSQNPDAQPHQLIPS
LVVHADTSSFINLSSNSSKDK*
>itf09g07000.t1.
MQDMTQSLPPGFRFHPTDEELITCYLMKKVASSADPTASMIAEADIYKFNPWELPG
RGYFGEKEWFFFSPRDRKYPNGHRPNRAAASGYWKATGTDKPILSATASSSSRCIG
VKKALVFYRGRPQKGIKTDWTMCEYRLLNNAHQQAFRQKGSMRLDDWVLCRVR
NKSNNVAEGRFGNNGKEDIITEKMNPSYDHHCYAGESQVLDGVEYFGHFQFQTGS
SSNHSNPQPNIMGGSVKEALENIKRVLLFEPCINGQPQILNQHESELNPTS*
>itf09g10480.t1.
METVPETASFDPLASHGGADTGQAAEAAVVYLPPGFRFHPTDEEIIVDYLKKKVTD
IAFSSVAIGEVDLNKCEPWDLPRKAKMMGKKEWFFFWQKDRKYPTGTRTNRATE
SGYWKATGKDKEIFQSGRGSSSSGVVVGMKKTLVFYKGRAPKGQKTNWIMHEYR
LHGNQFYNTTREEWVVCRVFHKQTTGVVYMRRSPPRNDAVSRIDSISIADHLLQTP
SKLPPLTDFPDYSDDKPPAPPLLPCSLTLQNPPSAVFYPPNPNSDTATAALKAESVS
QDAAGVVSPDVTPTEISWELPDGGGALGGLYDDSMADLDRYYSFLNYY*
>itf09g13340.t1.
MSESSLPPGYRFYPTEEELVSFYLRNKLQGARDEIHTVIPVVNIYDYNPWDLPQLA
GEYSRSDPEQWFFFIPRQDREARGGRPTRLTAEGYWKATGSPGCVYSVNNRIVGG
KRTMVFYRGRAPNGRKTEWKMNEYRAIEEEASQSSIANTANLKLHQEFSLCRVYK
RAKCVRAFDRRPTSSGPAITAAAFQPSPPQAAYYNLQGPSTSQQITAPPQAAAAGD
NQRSSSSSGGDLDSSLDFPPYAFSWDEFDELHCLLGNAQ*
>itf09g25220.t1.
MRGASLPPGFRFHPTDDELIGYYLKRKTEGLEIELEVIPVVDLYKFDPWELPEKSFL
PNRDMEWFFFCPRDKKYPNGSRTNRATKSGYWKATGKDRKVVCKPSTVGYRKTL
VFYRGRAPLGDRSDWVMHEYRISDYDSQGNPIFQGQFVLCRVIKRNDVSLKTINA
RGETSEKVRCNSSNGDISSVPDEPIVISGNISTQTTCKTTESNYSIPVPSSPYKTSQLG
DYEFHVHPSSSSLLISPDMILNSSKDYRHQGQHTFGYYPQYGFSNSTLWQPYENPE
LSSSSSYSSIRAEVELSDDLTRYGCTSAYSAQGSYIGFYGSEGASDQGYDQSILLTNP
NLF*
>itf09g26910.t1.
MMMMQGGAGNSESGQVCVPPGFRFHPTDEELLYYYLRKKVCYEAIDLDVIREVD
LNKLEPWDLKEKCRIGTGPQNEWYFFSHKDKKYPTGSRTNRATAAGFWKATGRD
KPIYHHHIPTINSSMSMSSRRIGMRKTLVFYTGRAPHGQKTDWIMHEYRLIIDNTNL
HAHSLLEEDGWVVCRVFRKKNHSRCSLQPEIIMSPGLQQQDDDDDECLAAVQMN
MKKKASCPSSGELPQQQHSRLPEEEEEAANNNNNSFGWYQQLWDNIGCNYMHLP
QLLSSESTSHHHTSPFLAPAAAPFTNMETSVPHGALQTFLRPHHHHNNKLNNPGGG

GGGDWAFLGKLLASNHRTNDDIGDQTTYCNLLLSQAAAADNMINIHHLSSTSTST
QQCFPFQHNAAASFDP*
>itf10g00780.t1.
MEGELKEESLPPGFRFHPTDEELITFYLVNKISDAGFTARAIGDVDLNKCEPWDLPG
KAKMGEKEWYFFSLRDRKYPTGVRTNRATNTGYWKTTGKDKEIFNSSTSELVGM
KKTLVFYRGRAPRGEKTNWVMHEYRIHSKSSYRTNKQDEWVVCRVFQKSAGAK
KYPSNHSRAVNPYTLEIGPSMMSSQMIPPDHGFHFAMAAPPGRNPAAAAAYITPAE
MQEFNRVFRGGGGATSAAINFPIHQPHQMNYPMGPPGAAGAGTGCFTISGLNLNL
GGSGGSASSQAVLRPMPPPPTPPTAMAQADVLTSCEAAGYVGGDMGNPANRYMG
MDQCADLDNYWPTY*
>itf10g07790.t1.
MARQAWLVDPNRLATKILSASSDPKRTNWKSNPSKACPNCQYSIDNSDVSHEWPG
LPRGVKFDPSDQEIVWHLIAKIGAEDLQPHPFIDEFIPTVDEDDGICYTHPQNLPGIK
MDGSCSHFFHRAIKAYNTGTRKRRKIHGDNFGDVRWHKTGRTKPVLLDGVQKGC
KKIMVLYVSPAKGGKPEKTNWVMHQYHLGTSEDEREGEYIISKVYYQQQQVKHN
KKSECQSPNGHECLTVKVDPVTPKSVTPEPPFTERRFSSSDAVSLTPAAYSSSNIQE
VNYIEDHMETPFQKSGNHDLITENQIDEMEAKNENEAGDDPKWYDLESQNIMDSQ
QLAEYISLCDEFIISQSPSREDQQNHKEKKRKTRLSDHYACLGAEDLKKDLEECQD
NEQQNHKEKKCKTRLSDHYACLGAEDSKKDLEECQDYELDPANIILDTPPDFRLSQ
LEFGSQDSFIAWGGHKIGSEEQGD*
>itf10g16540.t1.
MNLSVNGQSQVPPGFRFHPTEEELLHYYLRKKVTSEKIDLDVIRDVDLNKLEPWDI
QGNVAMCVSEKCKIGSGPQNDWYFFSHKDKKYPTGTRTNRATAAGFWKATGRD
KVIYSNCKRIGMRKTLVFYKGRAPHGQKSDWIMHEYRLDDNSPEPTGTSLVGELN
SAPEEGWVVCRVFKKKNYHKALESPHTVVQTHPGNDGVLDQILTYMGRSSSSSKQ
QNGAKNGNLIINGDDTPMQFSHAIQAARFLHLPPLETQSAMEILSSPTHASFEDMLA
EPEPSCNNTEPVDDMKTGPGPADWVALDRLVASQLNGQIESSKSYVTDDLSDGLY
FPIVDHHRPFGRPNNHHHNSQVNAGNEVEFWSYTNSSSSPSDPLSHLSV*
>itf10g16720.t1.
MSGVSMDALPLGFRFRPTDQELISHYLRRKINGRHSEVQVIPEVDVCKWEPWDLPS
LSVIKTDDPEWFFFCPLDRKYPNGTRCNRATEAGYWKATGKDRAIKTRKSTGSDQ
SNIPLIGIKKTLVFYKGRAPKGERTNWIMHEYRATEPDLDGTHPGQGAFVLCRLFH
KSDDKKCDEAEPSGSSPATNKSSPDDASSNLFQEAALLDRQPEKANASSIVMLPVE
SRMSDAVEHSTEEIKNEAPVGVDMMFNESKHESDSCTDIGLHISSPFPDDFGNDHN
GLDFQDGTCEQDVSLSELLGHFQGHENYSSEETTCQNNLSSERLILQDTLLEQTSPA
PFQTQVPPNDPDSILHTDDTHTHTNPVGHASDVAGGSGLRIRTRKRQNRPTSGNIIT
RGTAPRRIHLSLEQEPISVRSANVAEAGSFGSEVHEVQSLVAEVQASPISFKTKASEE
SYGDNSATNHRTTVDGETGTKVWMPQLFNQASSEHNVSQVSAAKRILQMEQVTV
SVSSGKAGTGHGRRAVIGEAHEGKECTSSTDEGEMPSSSTGSRETTQEHDTTVRVL
SKQGSNHHLSKIGFSFSSLVSPVLCGLSFSRLYAVSIYVIVAVSIFCVMIWKYPSKSV
VQR*
>itf10g20230.t1.
MESTADSRSDSGHPRLPPGFRFHPTDEELVVHYLKKKTASLPLPVTIIAEVDLYKFD
PWELPSKAKFGEQEWYFFSPRDRKYPNGTRPNRAATSGYWKATGTDKPILTCNGT

QKAGVKKALVFYRGKPPKGMKTNWVMHEYRLPDNVVNSMAPSSAHRPLGDDIV
NKKSSLRLDDWVLCRIFKKNGSSRPVESGGRGDPTEDATVATSTSLDGCGGAGDH
QNPNSRASRGLSFGEFFEINNGHNLYTGTMMSGGGINGTQTSWDSPRDQTCMAM
GITAAPPRIDEDHHHEAAAN MEGSTSFISLLNQLSPKMEINQNHSLRLMPK*
>itf10g22560.t1.
MGDNNNVNLPPGFRFYPTDEELVVHFLHRKAALLPCHPDVIPDLDLYPYDPWDLD
GKAMAEGRKWYFYSRRTSRRMTENGFWKPLGVEEPIFSSSGAGQKVGMKKYYAF
YVGEPPEGDKTDWVMQEYRLSDYSASTSSRSSSRRNRHSKIDYSKWVICRVFEGN
YDNGGGGDDGTELSCLDEVFLSLDDLDEISLPH*
>itf11g03560.t1.
MEDLPTGFRFYPTEEELVSFYLRNKLRGERPDIDLVIPVVNIYLHRPWELPQLAGVV
GGHGDYEQWFYFVPGPENIARGGKPNRLTTEGYWKATGSPGLVFSLNNRVIGEKR
TMVFYTGRAHNGTKTEWKINEYKAVRGDASASTPLSNLELCQEFSLCRLYKKSKC
DRAFDRRPLDLTATRRRIAQPPPQDNVQTTPITVQDPSATESTGAGKISPPGAAVIPS
NNATTTPLYWDNFEPIWDWEDCFSLL*
>itf11g07600.t1.
MELQPPQDGAGVVAPPIPKRAVAPPTSLAPGFRFHPTDEELVRYYLRRKACGKPFR
FQAVSEIDVYKSEPWELAEYSSLKNRDLEWYFFSPVDRKYGNGSRLNRATGKGY
WKATGKDRSVRHKSQTIGMKKTLVFHSGRAPDGKRTNWVMHEYRLADEELVKA
GVVQDAFVLCRIFQKSGLGPPNGDRYAPFIEEEWDDDTALVIPGGEAEDDAANGD
DAHVEGNELDQAAALCKAPQSPVEPQGLPFVCKRERSEDPEPLSLAQAKRSKHDD
PSSSNANGSEDSTTIQEPPTAMMTTTTTTNYSPALLEFPLLGSIEPKESHPTNVPTFD
SSTLEKSVPPGYLKFISNLENEILNVSMERETLKIEVMRAQAMINILQSRIDLLNKEN
EDLRRVVRGG*
>itf11g11490.t1.
MAPVGLPPGFRFHPTDEELVNYYLKRKIHGLEIELDIIPEVDLYKCEPWELAEKSFL
PSRDPEWYFFGPRDRKYPNGFRTNRATRAGYWKSTGKDRRVSSQNRAIGMKKTL
VYYRGRAPQGIRTDWVMHEYRLDDKECDDSSAGIQVDSYALCRVFKKNGICSEIE
EQAQQPSCNIQILDYSSAQTVANEYETPSPEVPFTSSSCVEEEEKEDKDDSWMQFIT
EDAWCSFTSPYATEEVSQTTFTN*
>itf11g18490.t1.
MDGRLRLHFVRDDGEVVKLPPGFRFQPTEQEIVFQYLQRKIFSLSLPASVIPELPNIC
RYDPWNLPGDMEQEDRYFFSSKEATKYPNGKQTSRVSSNGYWKPSSSEEKHITCP
GKSSSSIPMIMGMKKTLVFYYGKPPHGSKTNWIMHQYRLVAISPAGNPPLPPYFRH
AMKKDHSQGSLMQQIGNLVICHVFQKKRVNGKAGEESAEVCEDNINSSINEDENV
DRYNLMMRDGVSSSSCSDDGDSCVSSCSSSRVLNDEVSSSYSCGTLGYGNHQDAS
YI*
>itf12g00300.t1.
MDIQPVACVSGDDVQNPTDNDVDDHLLRDINDDDYFRLLPPGYVFNPTDEELIGE
YLDKQVKNLPLPRNRMNSVNLYSFNPEALCQMFKDYCGNKEWYFFTPRSRKYRN
GKRPNRAAGNGYWKATGADRVILASESNTKVGCRKALVFYMGKPPNGQKTPWI
MHEYRVEDGPECDRSVDAMKLDDWVLCRIYNKMDKSSKNNNNSSSTNKEEKPQE
KDEDDEEEEEEDKDAEIDDDVQENTTIATSDDQNHFEASPSYDHHQLLPIHIITPPLR
ATSPYNPHMWNQPMIPPQTSTSNNNVIQIQMTSFPNQNQQQIISGLQWPQHPPPHP

YEENLQQQEDHNNVLVHHHHNNPLPQNYFHPENTTFQALQPSINANLHHLPYQPH
NWQEPHPSFNGDHHLYYHHNSSAPITADNNIPYQHQNSELLEAPQPGINGDTNNPH
QQQNYSQAPQPSTNNGNDTTKTQTAAADDDEENDANYDTNQFLAD*
>itf12g02830.t1.
MIMADAVADESCAVPPGFRFHPTDEELVGYYLRKKIASQRIDLDVIRDIDLYRIEPW
DLQYKCRIGYEEQNEWYFFSHKDKKYPTGTRTNRATMAGFWKATGRDKAIYHKS
KIIGMRKTLVFYKGRAPNGRKTDWIMHEYRLESQDNNSPPQEEGWVVCRAFKKR
MAGSQARSNNAETWESSRKPSNETSVIMDPVDYYITTTRQTTPTSFISSHNHHSFLR
SKQELSSCNNLTDQFIHLPQLQSPSHPPPLQNPATSPSVPPESYYKFHGSNIEEDDEN
NNRVSDWRALDKLVASQLNHDDNPDDDQKRCNGAGAGGEPRLKNQDDSDVGLL
LLQSGGIWEDGEDDDINRFFSSNSDCNNIGICLFDK*
>itf12g06530.t1.
MSGFSGSDNDMDLPPGFRFHPTDEELITHYLTPKVLDTRFSAAAIAEVDLNSVEPW
DLPWKAKMGEKEWYFFCVRDRKYPTGMRTNRATGAGYWKATGKDKEIFKAKTL
VGMKKTLVFYKGRAPRGENTSWVMHEYYRLEGMHNLPKSAKNEWVICRVFKKS
SGGKKVPISGLIGGENGGENSKMMPPLMDISQASHVPCFSNSVEDQKPRNGESSGT
LSNHQIMPELDVDSGMMMMQDHSILKLLMENNQNSELGGLGPCDDDQQFLITSA
AGAVDLDCLWNYHY*
>itf12g06670.t1.
MGSIMDDKIIDDVMLPGFRFHPTDEELVGFYLKRKIQQRPLPIELIKQVDIYKYDPW
DLPKLATTGEKEWYFYCPRDRKYRNSARPNRVTGAGFWKATGTDRPIYSSEGTKC
IGLKKSLVFYRGRAAKGLKTDWMMHEFRLPTSPAPPPPPSNKFSGHNNFPPNDSW
AICRIFKKTSSMANRANFAHPWVNNILSEATPEALFNQFGSENIIMSSSSSAITPLQLS
TNNELQHFSAPPLNNIIPTYKPSSSSLFSNHHSPSPSSTPSPELPSCSNFMAPSSSSSLS
QAASVLFDVTPPTLFSPLNNTATSDHFEQSQHMEDDDEVVGLGNIRAMGFPFSLAS
EPWKQSTFSWDSPTY*
>itf12g12810.t1.
METLETGKEALVGQGDENERDNKDDLLGLPPGFRFHPTDEEIILHYLLEKVANNGF
VAIAIGEVDLNKVEPWDLPKKAKMGEKEWWFFCQRDRKYPTGMRTNRATESGY
WKATGKDKEIFKGKSNILIGMKKTLVFYRGRAPKGEKSNWVMHEYRLDGQFSYS
KTIRDAEWVVSRVFHKSAGVVRGIPMENNDNHPMDSFVDNLIHSPSAAATTTLPSL
RDLSPASNQNYFLENDDDNQNFKPLMASSSHGKSPYFPTTYPHSQILPPAAASVFN
NGLFYSPFLPNSPALKMEQTSYNNVSGSQETGLSMEVMMPANTETTSALSKLKQII
ATDDQTSPRPFDDLDTDPSYHDGDMDDHLDPYSFWDDYGPADP*
>itf12g15230.t1.
MKNLCVNGESQVVPPGFRFHPTEEELLQYYLRKKIASQKIDLDVIPDVDLNKLEPW
DIQEKCRMGLTPEKEKEWYLFSQSDKKYPSGSRRNRATSGGFWKATGRDKVIYGN
SRRIIGMRKTLVFYKGRAPRGQKSDWIMHEYRLELDNNHTTSHDGWVVCRLFKK
KNMHQKAFGQ*
>itf12g24590.t1.
MNSFVHVPPGFRFHPTDEELVDYYLRKKITSRKIDLDVIKDVDLYKIEPWDLQELC
RIGTEEQNEWYFFSHKDKKYPTGTRTNRATAAGFWKATGRDKAIYSKHDLIGMR
KTLVFYKGRAPNGQKSDWIMHEYRLESDPNGTPQEEGWVVCRVFKKRIATMRRE
SEHDSPIWYDDQVSFMPEMDSPTQQHPHTSSGGRSSYAQFPYRCKKELDLQYPPV

HAAAGDHPQFLQLPLLESPNKLLHAPPGLTYAPIPAFSGGHNIIHPMYVDNNNNNIL
GGHEHAAADHVTDWRVLDKFVASQLSQEELASKGPTTNYANAGDIFQTSDELNK
QNPAPENAPTSSSTSHHHQIDLWK*
>itf12g26670.t1.
MADTHLPIFSLPRSPSLYIPASPMTVIPLEESPATVLPPLNSLPVGFRFHPTDEELVDH
YLKLKINGSKTLASVIREIDICKLEPWDLPDLSMIKSYDNEWFFFCPIDRKYQNGQR
MNRATERGYWKATGKDRNILTKKRVKIGMKKTLVYYEGRAPDGKRSNWVIHEY
RATDKALDGTQPGQTPFVLCRLFKKNELKQDENAESPNSNGVEPNVSSPTVVKSP
GEDDQLEAVMPMGSDHIKAQPLTPEKSSVEESPVAQLPVDSNSISSNANNADEDLE
ILHDPDLDELCANLYEPSEEHLDIVSLPQTETQGLGSSYTYGAITNNCGDDMDIQSL
SETSVSDATEFMNSFLVSSDEVPYGDSGQQILPIEHATPNYVSTINKPSEAEMMQGL
VKTGFLENVPANALLQRHIEHVPNPSGGVYTPHTFNGGPEMWNSDPLNNSYLGQD
AFSTMCDGSQATNMVNLEGEGGSVNAVSSFGPGIRLRTRQARSQAADQLFTMQG
TAHRRIRLQMSFPAASVQSRLPEDSNQAESEKSAVTEGETIDSTATSSLETRDIIARE
FKRDGEISKNISTKTKGKGGFAACSNGNTISVFSKTGLLHSVYMPKVLIAVSLLIVL
VGSLACIRL*
>itf12g27410.t1
MKNNGGILNNNEQQQQQQLEMPPGFRFHPTDEELVVHYLCRKCASQSISVPIIAEID
LYKFDPWQLPDMALYGEKEWYFFSPRDRKYPNGSRPNRAAGTGYWKATGADKP
VGKPKTLGIKKALVFYAGKAPKGVKTNWIMHEYRLANVDRSAGKKNNLRLDDW
VLCRIYNKKGTVEKYYNAVDQNDVVLPKVEDQKPNITQFSQTATMLKQAALPPM
VQNQHDYMHFDTSESVLRWHTDSSCCSEQVLSSPEFAADKEVQSAPKWDDLDFQ
LNNFIDDPFQVPQYNDPFQDMFTYMHKPF*
>itf12g27600.t1
MANTTLFPGFRFHPTDVELMYCLKHKVIGRELPSEVKRAIPELNVRLYHPSELREK
SSSKDRMWYFFCPIEKKYASGSKLKRATETGYWKSTGGDRTVTYKGRTVGKIKTL
IFHQGHSGKGERTDWVIHEYRMEDKHLADARIVQDSYVICKVFKKTGPGPQNGSQ
YGSFEEDWDYDDVCAEPDLSGVSPAMPASTDCQSCPVGIESTSSFALVEPGPSSAEP
GSSLAEPHINELSEGGDINELLGPFFMEHNGLLPIESNNNENTSDLTQARCVEIAECN
DGDDIYNDLGGLGNLPMLGEDGFSNILYPLEETTAYYGYTDAYINQARSAEIAECN
MTAAAAAAAAYFGDSLGGLGNLPTLGEDGFGDTAGYVELDDLLKPLDESNCGSE
TQHSYPSYNNNVAIDVEQSHIRGNTTSGSNEFDPSSATRGVHHIQKGTIGAYNSW*
>itf13g04700.t1.
MNSLSHVPPGFRFHPTDEELVDYYLRKKVALKRIDLDVIKEIDLYNIEPWDLQELC
KIGCEEQSDWYFFSHKDKKYPTGTRTNRATKAGFWKATGRDKAIYSKHCLIGMR
KTLVFYKGRAPNGLKSDWIMHEYRLETNENGAPQEEGWVVCRAFKKRMPTMAR
KEGADHESPLCSNGWYDDQISSFIPSDFESPRRISTHHHHHPNYNNINPPPYNMNPH
FLNSCKQELHHHQLHYNIIVPHEHEHHPPFIHQSSSSSLPQLESPKLPHPTPQFHMIN
SSSSSSPSLLYCNDQSVADDHDHLATDWRILDKFVASQLMSHDEEDNNNSNGDGK
ETSLRASSSFQP*
>itf13g16970.t1.
MEKLSFVKNGVLRLPPGFRFHPTDEELVVQYLKCKVLSCPLPASIIPEVDICKSDPW
DLPGDLEQERYFFSTREVKYPNGNRSNRATSSGYWKATGLDKQIVSSKGHRQLVG
MKKTLVFYKGKPPQGARTDWIMHEYRLTNAESLPNNPPQENWVLCRIFLKKRGA

KNEEENTPAQAQTPTQAQAVTNNRGAGLKNNSKPVFYDFLARERTDLNLAPASSS
SGSSGVTEASCNDKTDDHEESSSCCNSFTTFRTKP*
>itf13g22540.t1.
MARPSWLVDSKRIATKIRSASDDPGNINWKSNPTRTCPSCHYIIDNSDETHEWPGLP
RGVKFDPSDQEIIWHLLAKVGLNDLKSHPFIDEFIPTVNEDDGICYTHPQNLPGVKQ
DGTVSHFFHRAIKAYNTGTRKRRKIHGDNFGDVRWHKTGRTKPVVLDGIQRGCK
KIMVLYVSSAKGGKAEKTNWVMHQYHLGTGEDEREGEYVVSKVFYQQQQVKHS
EKNELESPEETECLDAKKDPSTPKTVTPEPLHTETRPSSFVAGLETPIPYISSNVQHR
EVDYTEADMEIPLEKADNQVEITQNQTEQMELQNENEAGEERKWWDSESQNLLD
SQQLVEGLSLCDELLQSQSPNRDGDQNGKEHKSTSCLSDYRHLGPENFKKDLEEC
QELVLDVPPANQSGKEKKCMPHLSDYGHLGTENYKLLENSQELVLDPENIMLDTP
PDFRLSQLEFASQDSYIAWGGNNIDSTEQGS*
>itf14g00550.t1.
MEDYPQGFRFSPTEEELVCFYLKHKLQGDREDIDAVIPVVNIYDHCPWNLPQLVGE
RCRGDDLEELFFFVDMQENISRGGKPKRLTPQGYWKAAGIPALMYSNNNEIIGGK
RTMIFYRGRAPTGIKTEWKMIEYKAILGQPPRTATMSDVRLGHEFSLCRIYKKARF
DRGFDRRPSAPAAIFAVPRAPEPPSPHLQTVPEAIAAQASSSNPQAVPESTEDPSSSQ
NNFWDVCVPLWDWADMGF*
>itf14g12330.t1
MADGGDISLSRQNEEDNSMERISGINNNNNNDETNGKIEISLATASSMFPGFRFSPT
DEELISYYLKKKLEAFDECVEVIPEVEIWRHEPWDLPAKSVVQSDNEWFFFSPRGR
KYPNGSQSRRATESGYWKATGKERNVKSGSKVIGTKRTLVFHTGRAPKGQRTQWI
MHEYCIGEKEYQDSMVVCRLRKNNEFHLNDTLGNSRNQSIVNTSNNAFSELEYTG
SLGGLNAGDSCSKECSSSLNSHSVEQIDPGVDCDLVNEISQCGSSSHQKDDGNAED
WFADIMRDDIVKLDDTSLNTSLDVLPVRNKNPVPDIKPKQPAQGLKPHVLPFQGTA
NRRLRLRRDRVTFFETSWIAQLFSQHGAKVLIADTRDHESQSICKDLGPGNAFFVH
FDVTSESDVQNAVNKAVSTHGRIDIMVNNAGIVGDKMKRSFLGLAEDDKLDMMT
EDVAEAVLYLASDESKYVSGHNLVVDAGFSISNLAVNLFNQ*
>itf14g17960.t1.
MGLRDIEATLPPGFRFYPSDEELVCHYLYKKVVANNQRLSKYTLVEIDLHTCEPW
QLPDVAKLNSKEWYFFSFRDRKYATGYRSNRATTCGYWKGTGKDRRILDPATGT
VLGMRKTLVFYHHRAPHGVKTDWIMHEFRLENPHIPPKEDWVLCRVFQKSKAAG
VCDESIVGAAAGASNSSPNAVNNNGFLPAGYSPMSGVPAGNVGMAQSKREEDDE
YGFFFNVNCEEPNFEAAGGGVPLWLDEDFRFDNGGNGLLFI*
>itf15g01560.t1.
MEEEDFPPPGYRFFPTEEELVSFYLKHKLEGRFQEHIDVVIPVLNIYDHDPWSLPQF
AGKYNRSDPEQWFFFIPMQEREARGGRPTRLTAGGYWKATGSPGLVYSGNNRVIG
GKRTMVFYTGRAPNGRKTEWKMNEYRFTERDASNSPQEFILCRVYKKPKCARAF
DRRPACVAPAAPPAVDGGELLALQPPPPPQVAAAANDASASTSQQKAEADKKSSS
QETSSPSPPHGGDNVEDEPVWDFSDMILWEWDPFKVLYN*
>itf15g03430.t1.
MEKEEATLTMEVEKGLRDRYFKSLPPGIRFRPTDVELMDYLKKKVLKERMPLHRI
KTCNIYKYHPNDLCRPDDLAREKESYFFTSRDKKYPNGARPDRKAGDGFWKATG
RDMPISCKGKVVGSKKTLVYYEGKHKDVSLHKTNWIMHEYTINPNIKITPVAQGQ

SDNNNNSDNNILDECVLAKIYEKTTGSKKGADNCSPSQDDTQQILDSEQLPPEEQD
NPILEPQEYPTAEQVAPPPQNPNHQNPTAETGGGGGYNTNMMILQGSEQLPLPPPE
AQNNPILEPQQYSRATHMGSFTGPNNEVRAQNNPILEPQQYFRATHMGSLTGPNN
EVRAQNNPILEPQQYPMATCMDSLTGSRGGVRVQNNPILEPQQYPMATHMGSLIG
PNNEIRAQNPILEPQQYPTANRMDSLTGPKCEVRTQNNPILKPQQYPATTHMNSLT
GHNREILKEDYSTALQAAAAAAASYPNHQNPMAGAAAAAAYHRYMMIPVSEQPP
LPPPEAQENPILEQVAELPPFPYQENPMDVAAAASATFYSWDAGSYGYESLQTDEN
FYGINSGAFSFEENNNGFWGSLNGGLEDVVNMYIGNNVNNPSANPHGSHGQANN
DGDNVNPSHNPHGPANVSDK*

>itf15g08990.t1.

MAASCSAQRPNGYGDDDSDEWKRSLPPGHGFYPTDQELITKYLKHKAQNRKIHA
GIINDLDIYNYHPEELEAANALNNWNGRRYFFTAFKRKTKDGTRGDRAVGGGKG
YWKASQAREPLKDGGGVVIGTKQPLVFHDGEGKKTSWLMSEFRYPGNVDPFPLY
NETEHELALCVIYYHGERKIVGDNQESKSNSTNSMENLQSSHLIPTPCANPNFASTS
TMQNPNHPPYPILNPNFNATSTLQSPNHPPSPAQNSNFITISSPYPTQNPNFNATSTL
QSHNHPPLPCQNSNFIATSSLYPTQNLNFNSTSALQSPNHPPSPTQNSNFIATSSLYST
QNPNLNATFALQNPNHSPPLTQNSNFIPTSSMQNLNHSLSFAQNPNFIVTSSLRNSN
HPVSSSQNPNFIATSSQQNPTFSQSSLAQNSFLQDAWNWNELPTPLFIDEETIHQYF
NDDGNGDTFQLRVLSYDSDDTDESSSRKKRIKKKKSLK*

>itf15g15530.t1.

MAVLPGKMSQQNQDERCQKNIEGEETADEHEHDMVMPGFRFHPTEEELVEFYLR
RKVEGKRFNVELITFLDLYRYDPWELPALAAIGEKEWFFYVPRDRKYRNGDRPNR
VTTSGYWKATGADRMIRGENSRSIGLKKTLVFYSGKAPKGIRTSWIMNEYRLPHH
ETERLQKAEISLCRVYKRAGVDDHHPSLPRSLQTSRAATATAAASSSSSRGTAAIK
KLHESSFSPYQVAEEKISAETSGSSSTEVGTSLGLSSYQSSYAAPIVPAPPSPAFFSAS
SSAVAVADDLHRLVNSQQQISLQHPQLFQSLSSPFPPPPMLQPLPPQNLALPPGSLH
QAAAAFSDRLWDWSSMGDASRDYGAGNPFK*

>Prupe.5G006200.1

MLGMEDAVMSELSGEDINEQGLPPGFRFHPTDEELITFYLASKVYNGSFCGVDIAE
VDLNRCEPWELPDMAKMGDREWYFFSLRDRKYPTGLRTNRATGAGYWKATGKD
RQVYSASTGALLGMKKTLVFYKGRAPRGHKTKWVMHEYRLHGHLSSYGGHACK
DEWVICRINHKTGDKKIPLQLDQVEVEAASSNYNCLPPLLESPTAAAKPTFLQQGQ
CPHQSHNPMQMQSPHPLPPFLFRHQENDLKSLINPVVSQPHLFSSFPINALQTQSSFS
PTTTNSNTALLTNDKNPSPLQSLLFKSLFSSHDQDCNNTIPKQCKTEPNYFSHFQTP
ANNNNNNCDLNSLNLMEKNHHHLHQPNTPYHQYSNHPNDPLLFDCLDYSVLGFP
DAAGTATTVHEHDTCPSTAFNRAAFQTMLDLPPIKVTGESWPLDYIMDAK*

MYB Protein sequences for phylogenetic analysis and multiple sequence alignments

MYBs

>PpMYB10.1

MEGYNLGVRKGAWTREEDDLLRQCIENHGEGKWHQVPNKAGLNRCRKSCRLRW
MNYLKPNIKRGEFAEDEVDLIIRLHKLLGNRWSLIAGRLPGRTANDVKNYWNTRL

RTDSRLKKVKDKPQETIKTIVIRPQPRSFIKSSNCLSSKEPILDHIQTVENFSTPSQTSP
STKNGNDWWETFLDDEDVFERATCYGLALEEEEFTSFWVDDMPQSKRQCTNVSE
EGLGRGDFSFNVDFWNH
>MdMYB114
MISNLLGIMRKGAWTQQEDDILRQYVEKHGDGKWHQVPRETGLNRCRKSCRLRW
LNYLKPNLKSGDFTEDEIDLIHRLQKLLGNRQVPLFGKQKKNRQVILYSKQKPNAC
MHELVIIKILLTLKIVSKQKNFIRSWESLPRHISQ
>MdMYB10
sMEGYNENLSVRKGAWTREEDNLLRQCVEIHGEGKWNQVSYKAGLNRCRKSCRQ
RWLNYLKPNIKRGDFKEDEVDLIIRLHRLLGNRWSLIARRLPGRTANAVKNYWNT
RLRIDSRMKTVKNKSQEMRKTNVIRPQPQKFNRSSYYLSSKEPILDHIQSAEDLSTP
PQTSSSTKNGNDWWETLLEGEDTFERAAYPSIELEEELFTSFWFDDRLSPRSCANFP
EGQSRSEFSFSTDLWNHSKEE
>PcMYB10
MEGYNVNLSVRKGAWTREEDNLLRQCIEIHGEGKWNQVSYKAGLNRCRKSCRQR
WLNYLKPNIKRGDFKEDEVDLILRLHRLLGNRWSLIARRLPGRTANDVKNYWYTR
LRIDSRMKTVKNKSQETRKTNVIRPQPQKFIKSSYYLSSKEPILEHIQSAEDLSTPSQ
TSSSTKNGNDWWETLFEGEDTFERAACPSIELEEELFTSFWFDDRLSARSCANFPEE
GQSRSEFSFSMDLWNHSKEE
>AtPAP1
MEGSSKGLRKGAWTTEEDSLLRQCINKYGEGKWHQVPVRAGLNRCRKSCRLRWL
NYLKPSIKRGKLSSDEVDLLLRLHRLLGNRWSLIAGRLPGRTANDVKNYWNTHLS
KKHEPCCKIKMKKRDITPIPTTPALKNNVYKPRPRSFTVNNDCNHLNAPPKVDVNP
PCLGLNINNVCDNSIIYNKDKKKDQLVNNLIDGDNMWLEKFLEESQEVDILVPEAT
TTEKGDTLAFDVDQLWSLFDGETVKFD
>AtMYB114
MEGSSKGLRKGAWTAEEDSLLRQCIGKYGEGKWHQVPLRAGLNRCRKSCRLRWL
NYLKPSIKRGKFSSDEVDLLLRLHKLLGNRWSLIAGRLPGRTANDVKNYWNTHLS
KKHEPCCKTKIKRINIITPPNTPAQKVDIF
>PyMYB114
MEDSNLLGIMRKGAWTQQEDDILRQCVEKHGDGKWHQVPRETGLNRCRKSCRQ
RWLNYLKPNLKSGDFTEDEIDLIHRLQKLLGNRWSIIAGRLPGRTAGKVKNYWNS
KQRKELEYMKDKSKERTKATSVIRPQPRRARVAIFQSEENRSRL-
QTSSPPTESAIDSWKTMLHDTDNVDGTPFSSLGLGEDLFTNFWVEDIAQSTMVGM
NSADEGLHMSGNFSFRENLWNLEEEITKI
>AcMYB110
METVPLGVRKGAWTEEEDKLLKKCIEKYGEGKWYQIPLRAGLNRCRKSCRLRWL
NYLRPNINRGIFTADEVDLIIRLHKLLGNRWSLIAGRLPGRTSNDVKNYWNTHLQK
KLISTREEPIPKTQKTIVPKVTRPQPRIFMKHQPLLEGKTVIADSIQPRDVNLPKPSPT
PTPTNNEILWWDYKILGPEIDDMGINWSIDGSIFEEPILGIQSAGDRPLQQNQRDWS
DTVIEDVDLWNLLGDD
>MdMYB1
MEGYNENLSVRKGAWTREEDNLLRQCVEIHGEGKWNQVSYKAGLNRCRKSCRQ
RWLNYLKPNIKRGDFKEDEVDLIIRLHRLLGNRWSLIARRLPGRTANAVKNYWNT

RLRIDSRMKTVKNKSQEMRKTNVIRPQPQKFNRSSYYLSSKEPILDHIQSAEDLSTP
PQTSSSTKNGNDWWETLLEGEDTFERAAYPSIELEEELFTSFWFDDRLSPRSCANFP
EGQSRSEFSFSTDLWNHSKEE
>MdMYB110a
MEGCNVNLSVMRKGAWTREEDDLLRQCIEILGEGKWHQVPYKAGLNRCRKSCRL
RWLNYLKPNIKRGDFTEDEVDLIIRLHKLLGNRWSLIAGRLPGRTANDVKNYWNT
RLRINSRMKTLQNNSQETRKTIVIRPQPRSFIKSSNYLSSKEPIIDHIQSEEDLSTSSQT
SSLTNNGNDWWKTLLEDDDILLKELYVPVLS
>MdMYB110b
MEGCNVNLSVMRKGAWTREEDDLLRQCIEIHGEGKWRQLPNKAGLNTCRKSCRL
RWLNYLKPNIKRGDFTEDEVDLTIRLHKLLGNRWSLIAGRLPGRTANDVKNYWNT
QLRLNSRMKTVKNKSQEMRKNIVIRP
>AtMYB113
MGESPKGLRKGTWTTEEDILLRQCIDKYGEGKWHRVPLRTGLNRCRKSCRLRWL
NYLKPSIKRGKLCSDEVDLVLRLHKLLGNRWSLIAGRLPGRTANDVKNYWNTHLS
KKHDERCCKTKMINKNITSHPTSSAQKIDVLKPRPRSFSDKNSCNDVNILPKVDVV
PLHLGLNNNYVCESSITCNKDEQKDKLININLLDGDNMWWESLLEADVLGPEATE
TAKGVTLPLDFEQIWARFDEETLELN
>AtMYB75
MEGSSKGLRKGAWTTEEDSLLRQCINKYGEGKWHQVPVRAGLNRCRKSCRLRWL
NYLKPSIKRGKLSSDEVDLLLRLHRLLGNRWSLIAGRLPGRTANDVKNYWNTHLS
KKHEPCCKIKMKKRDITPIPTTPALKNNVYKPRPRSFTVNNDCNHLNAPPKVDVNP
PCLGLNINNVCDNSIIYNKDKKKDQLVNNLIDGDNMWLEKFLEESQEVDILVPEAT
TTEKGDTLAFDVDQLWSLFDGETVKFD
>AtMYB90
MEGSSKGLRKGAWTAEEDSLLRLCIDKYGEGKWHQVPLRAGLNRCRKSCRLRWL
NYLKPSIKRGRLSNDEVDLLLRLHKLLGNRWSLIAGRLPGRTANDVKNYWNTHLS
KKHESSCCKSKMKKKNIISPPTTPVQKIGVFKPRPRSFSVNNGCSHLNGLPEVDLIPS
CLGLKKNNVCENSITCNKDDEKDDFVNNLMNGDNMWLENLLGENQEADAIVPEA
TTAEHGATLAFDVEQLWSLFDGETVELD
>IbMYBb
MVGAAEKAGWRKGPWTPEEDKLLGDYVSLHGEGRWSSVARCAGLNRNGKSCRL
RWVNYLRPGLKRGHISPQEEGIIIELHALWGNKWSTIARYLPGRTDNEIKNYWRTH
FKKKPASGKTSEKQDRRKNRRKRNEEKVINDTKPQETMSNNDSSCITAAAAQMGG
TSANTTTTLYHEDIESWVDSFAMDMDGLWGGGLWNLDDDDSYPEAALLEQGHVI
QNPCGFGADHAVNLWNGGFIF
>IbMYB44
MASISPNGRRKDMDRVKGPWSPEEDELLQQLVQKHGPRNWSLISKSIPGRSGKSCR
LRWCNQLSPQVEHRAFTAEEDDTIIRAHARFGNKWATIARLLAGRTDNAIKNHWN
STLKRKCSSMSADEGNDLADRLQQQPLKRSVSAGAAVTLSGLHFNPGSPSGSDVS
ESSLPVMSPSHVFKPIARTGGVLPPPVETPPPPPPPPPANDPPTSLSLSLPGVDSSSDV
SPRLTESTQPISPIQLFSSAIHTPPPPPPLPVPFQQPLEKFDLGGGAPPPMACPIPPKEA
VPAPAQQDRVFLPFSQELLAVMQDMIKTEVRNYMMGVEPQQPQPSQQQRYHQHH

HQQHQQQQQFQFQQQQQLQNGIGRGMCLQRATSNDGLRYAAAATVNRVGVNRL
E*
>AtMYB44
MADRIKGPWSPEEDEQLRRLVVKYGPRNWTVISKSIPGRSGKSCRLRWCNQLSPQ
VEHRPFSAEEDETIARAHAQFGNKWATIARLLNGRTDNAVKNHWNSTLKRKCGG
YDHRGYDGSEDHRPVKRSVSAGSPPVVTGLYMSPGSPTGSDVSDSSTIPILPSVELF
KPVPRPGAVVLPLPIETSSSSDDPPTSLSLSLPGADVSEESNRSHESTNINNTTSSRHN
HNNTVSFMPFSGGFRGAIEEMGKSFPGNGGEFMAVVQEMIKAEVRSYMTEMQRN
NGGGFVGGFIDNGMIPMSQIGVGRIE
>AtMYB70
MSGSTRKEMDRIKGPWSPEEDDLLQSLVQKHGPRNWSLISKSIPGRSGKSCRLRWC
NQLSPEVEHRGFTAEEDDTIILAHARFGNKWATIARLLNGRTDNAIKNHWNSTLKR
KCSGGGGGGEEGQSCDFGGNGGYDGNLTDEKPLKRRASGGGGVVVVTALSPTGS
DVSEQSQSSGSVLPVSSSCHVFKPTARAGGVVIESSSPEEEEKDPMTCLRLSLPWVN
ESTTPPELFPVKREEEEEEKEREISGLGGDFMTVVQEMIKTEVRSYMADLQLGNGGG
AGGGASSCMVQGTNGRNVGFREFIGLGRIE
>AtMYB73
MSNPTRKNMERIKGPWSPEEDDLLQRLVQKHGPRNWSLISKSIPGRSGKSCRLRW
CNQLSPEVEHRAFSQEEDETIIRAHARFGNKWATISRLLNGRTDNAIKNHWNSTLK
RKCSVEGQSCDFGGNGGYDGNLGEEQPLKRTASGGGGVSTGLYMSPGSPSGSDVS
EQSSGGAHVFKPTVRSEVTASSSGEDPPTYLSLSLPWTDETVRVNEPVQLNQNTVM
DGGYTAELFPVRKEEQVEVEEEEAKGISGGFGGEFMTVVQEMIRTEVRSYMADLQ
RGNVGGSSSGGGGGGSCMPQSVNSRRVGFREFIVNQIGIGKME
>AtMYB77
MADRVKGPWSQEEDEQLRRMVEKYGPRNWSAISKSIPGRSGKSCRLRWCNQLSPE
VEHRPFSPEEDETIVTARAQFGNKWATIARLLNGRTDNAVKNHWNSTLKRKCSGG
VAVTTVTETEEDQDRPKKRRSVSFDSAFAPVDTGLYMSPESPNGIDVSDSSTIPSPSS
PVAQLFKPMPISGGFTVVPQPLPVEMSSSSEDPPTSLSLSLPGAENTSSSHNNNNNA
LMFPRFESQMKINVEERGEGRRGEFMTVVQEMIKAEVRSYMAEMQKTSGGFVVG
GLYESGGNGGFRDCGVITPKVE
>FaMYB1
MRKPCCEKTETTKGAWSIQEDQKLIDYIQKHGEGCWNSLPKAAGLRRCGKSCRLR
WINYLRPDLKRGSFSEDEEDLIIRLHKLLGNRWSLIAGRLPGRTDNEVKNYWNSHL
KKKILNTGTTLRPNKPRPEIKHAPYNKLVKYFNEMDDEVVDEVSSADSAAGCLVP
ELNLDLTLSIKTSTGMADPQVA
>FaMYB44.1
MAMNRKEMDRMDRIKGPWSPEEDDALQSLVQKHGPRNWSLISKAIPGRSGKSCR
LRWCNQLSPQVEHRAFTPEEDDTIIRAHARFGNKWATIARLLSGRTDNAIKNHWN
STLKRKCSSMSDDGNGNGVGYDGNFHEQPPLKRSVSAGSGVPVSTELFMNPGSPS
GSDLSDSSVQVMSPSHVYRPVARSGGVLPPVETTSSSNNNNINLNSDPPTSLSLSLP
GVDSCEVSNHRVAAVVESTQNQAAAQPAAYDPMRSFPAELLELVQGMIKKEVRS
YMEGLEQSGFGVMRQQSDGFRNVAVKRIGISRIE
>FaMYB44.2

MASSSTKKAAADRIKGPWSPEEDEALQRLVQSYGPRNWSLISKSIPGRSGKSCRLR
WCNQLSPEVEHRPFTPEEDDTIIRAHARFGNKWATISRLLNGRTDNAIKNHWNSTL
KRKFSSMSDDMSPEHQPLKRSASVGAGTTVSGLYLNPGSPSGSDLSDSSLPGVIPHS
QAAFRPVAMTVAPPVEPVALAVAVDPPTSLSLSLPGSNEGRTHQRSASGSGSSWIV
SPKPKVVHQPQVIQVQPQEAEAAAAAAAAAAAAARVGKQNSSEVGIDKQFFTAEF
LDVMQEMIRTEVRNYMSGIEHKGMMMHTEAAIKNAVVKRIGISKVE
>FaMYB44.3
MDCESVQQRELDRVKGPWSPEEDDLLRKLVQRHGARNWTLISKSIPGRSGKSCRL
RWCNQLSPEVEHRAFTAEEDEIIAAAHSKYGNKWATIARLLNGRTDNAIKNHWNS
TLKRKYSSMAEDGGGGGVEENQYHILRPEKKSATASVSGRGYSPTSPSGSDVSDSG
VAPAVTSAQVFWPMARAVMRTESPPPRNEPEPSTLLTLSLPGGSEVATRREEKTTS
LCLTTSLDENQNRTMSFGPEFMSLMQEMVRKEVRNYMDDKHLGIPKLS
>NtMYB44
MEAIKETGRIKGPWSPEEDELLQRLVEEHGPRNWSIISKSIPGRSGKSCRLRWCNQL
SPQVEHRPFTAEEDETILKAHAKLGNKWATIARFLSGRTDNAIKNHWNSTLKRKCP
STSDDLSFETPQQPLKRSSSIGPGTISMNPGSPSGSDLSDSGFPGIAQSNVYRPAARPS
GVLPPSVPDPPTFLSLSLPGSGFNEATGRVTPQVAEPDPAPSSAPGLMPQNLPVIEN
YSFGPSIMEKQLFSPEFLAVLQNMIHKEVKNYTEKGQKYP
>OsMYB44
MMASCRRGGGGDVDRIKGPWSPEEDEALQRLVGRHGARNWSLISKSIPGRSGKSC
RLRWCNQLSPQVEHRPFTPEEDDTILRAHARFGNKWATIARLLAGRTDNAIKNHW
NSTLKRKHHSSLLADDLRPLKRTTSDGHPTLSSAAAPGSPSGSDLSDSSHHSLPSQM
PSSPPHLLLPQHVYRPVARAGGVVVPPPPPPPPATSLSLSLSLPGLDHPHPDPSTPS
EPAVQLQPPPPSQMPPPTPSCVRQEPPQMPFQLQPPPPPRPSAPFSAEFLAMMQEMI
RIEVRNYMSGSAAVDPRSSPDNGVRAASRIMGMAKIE
>SlMYB44
MAAITQRKDSDRIKGPWSPEEDELLQTLVEKHGPRNWTLISKSVPGRSGKSCRLRW
CNQLSPQVEHRAFTPEEDDTIIRAHAKFGNKWATIARLLSGRTDNAIKNHWNSTLK
RKCPSMSEDLSFETPQPPLKRSSSVGPCTNFSSVMNPGSPSGSDLSDSSLSGFPQSHV
YRPVPRTGGIFPLPPPPPVKQIEIPPSVPDPPTSLCLSLPGSGSGSIEKPTQSPKSPPLPP
PPLPAVDKPIPPSVPVTCPPSAFMGHLAQSNQSYDFSAAPKSGEKQFFTPEFLSVLQ
DMIRKEVKSYMSGFEQNGLCMQTDAIRNAVIKRIGISKIE
>StMYB44
MAAITQRKDSDRIKGPWSPEEDELLQTLVEKHGPRNWTLISKSVPGRSGKSCRLRW
CNQLSPQVEHRAFTPEEDDTIIRAHAKYGNKWATIARLLSGRTDNAIKNHWNSTLK
RKCPSMSEDLSFETPQPPLKRSSSVGPCTNFSSVMNPGSPSGSDLSDSSLSGFPQPLV
YRPVPRTGGIFPLPPPPPPVKQIEIPSSVPDPPTSLCLSLPGSGSIEKPTQSPNSPPLPPP
PLPVVDKPIPPSAAVMGHLPRSNQSYDFCAAPKSGEKQFFTPEFLSVLQGMIRKEV
KSYMSGFEQNGICMQTDAIRNAVIKRIGISKIE
>IbMYB1
MVISSVWSGSSSRVRKGSWSEEEDQLLRECIQKYGEGKWHLIPLRAGLNRCRKSC
RLRWLNYLRPDIKRGEFSPDEIDLILRLHRLLGNRWSLIAGRIPGRTANDVKNLWN
THLQKKVSAMASSRQDNYWKGKAPEITENTVVRPRPRRFLKASSSPTTLLTGNAT

MVAYDGQLQEHMTTQPETTSDLLMENVQQKNLTTTLPSALETTPHDNVKWWED
VLSDKELNEEGQICWSEFPTDIDLLSELLS
